# Diagnostic significance of microRNAs in sepsis

**DOI:** 10.1371/journal.pone.0279726

**Published:** 2023-02-22

**Authors:** Xiaolan Zheng, Yue Zhang, Sha Lin, Yifei Li, Yimin Hua, Kaiyu Zhou

**Affiliations:** Key Laboratory of Birth Defects and Related Diseases of Women and Children of MOE, Department of Pediatrics, West China Second University Hospital, Sichuan University, Chengdu, China; Gifu University School of Medicine Graduate School of Medicine: Gifu Daigaku Igakubu Daigakuin Igakukei Kenkyuka, JAPAN

## Abstract

**Background:**

Sepsis is a life-threatening condition that induce tens of million death each year, yet early diagnosis remains a formidable challenge. Many studies have focused on the diagnostic accuracy of microRNAs (miRNAs) for sepsis in recent years, particularly miR-155-5p, miR-21, miR-223-3p, miR-146a, and miR-125a. Thus, we conducted this meta-analysis to explore if miRNAs may be used as a biomarker for sepsis detection.

**Methods:**

We searched PubMed, the Cochrane Central Register of Controlled Trials, EMBASE, and China National Knowledge Infrastructure through May 12, 2022. This meta-analysis was conducted using Meta-disc 1.4 and STATA 15.1 in a fixed/random-effect model.

**Results:**

The analysis included a total of 50 relevant studies. The overall performance of total miRNAs detection was: pooled sensitivity, 0.76 (95% confidence interval [CI], 0.75 to 0.77); pooled specificity, 0.77 (95%CI, 0.75 to 0.78); and area under the summary receiver operating characteristic curves value (SROC), 0.86. The subgroup analysis suggested that detection in miR-155-5p group had the highest area under the curve (AUC) of SROC among all miRNAs: pooled sensitivity, 0.71 (95%CI, 0.67 to 0.75); pooled specificity, 0.82 (95%CI, 0.76 to 0.86); and SROC, 0.85. MiR-21, miR-223-3p, miR-146a, and miR-125a had SROC values of 0.67, 0.78, 0.69, and 0.74, respectively. The specimen type was found to be a source of heterogeneity in the meta-regression study. The SROC of serum was higher than that of plasma (0.87 and 0.83, respectively).

**Conclusions:**

Our meta-analysis revealed that miRNAs, specifically miR-155-5p, could be useful biomarkers for detecting sepsis. A clinical serum specimen is also indicated for diagnostic purposes.

## Introduction

Sepsis is a life-threatening disease that induce about 11 million death each year [1]. The main cause of sepsis is the maladjustment of the host’s response to infection [2]. Sepsis is treatable and timely implementation of targeted interventions can improve outcomes [3]. Meanwhile, delayed diagnosis is associated with increased mortality [4]. Therefore, early diagnosis of sepsis is the key to improve the survival rate. Nevertheless, sepsis is a heterogeneous syndrome and the diagnosis of sepsis detection is mainly according to the site of infection, etiology, onset time, and the patient’s profile [2]. However, traditional screening methods and biomarkers such as C-reactive protein (CRP) and procalcitonin (PCT) lack specificity, which leads to the early diagnosis of sepsis is still a formidable challenge. Therefore, it is important to find novel and reliable biomarkers for early diagnosis of sepsis.

MicroRNAs (miRNAs) are a type of small noncoding RNA with an average length of 18–25 nucleotides [5]. Previous research [6, 7] has discovered that circulating miRNAs can be used as biomarkers to detect various diseases. Vasilescu et al. [8] were the first to discover that plasma miR-150 was a potential biomarker for sepsis. Numerous studies [9–12] have since confirmed the significance of miRNAs in sepsis. Several miRNAs have been mentioned in several studies with varying diagnostic effectiveness. Shen et al. [13] conducted a meta-analysis in 2020 to validate the diagnostic accuracy of miRNAs for sepsis, and found that miR-223-3p might be used as a sepsis indicator. In recent years, however, a growing number of research have focused on the diagnostic accuracy of miRNAs for sepsis, particularly miR-155-5p [14–16], miR-21 [9, 10, 17], miR-146a [18–20], and miR-125a [21–23], but the results have been inconsistent. Thus, we collected all published case-control articles to gather evidence on the diagnostic accuracy of miRNAs for sepsis.

## Materials and methods

### Study protocol

This analysis was carried out according to a predetermined protocol, as recommended by Deeks [24]. The Preferred Reporting Items for Systematic Reviews and Meta-Analyses (PRISMA) Statement (S1 Table) was used for data collection and analysis [25]. The ethics approval was not required because this was a comprehensive literature review. This study was registered with PROSPERO (CRD42022361151).

### Search strategy

To find relevant studies, we searched PubMed, China National Knowledge Infrastructure (CNKI), EMBASE, and the Cochrane Central Register of Controlled Trials (CENTRAL) databases until May 12, 2022. Keyword search terms were (‘sepsis’ OR ‘pyemia’ OR ‘septicemia’) AND (‘MicroRNAs’ OR ‘miRNAs’ OR ‘MicroRNA’ OR ‘miRNA’). PubMed database was searched as follows: (Sepsis[MeSH Terms] OR pyemia OR septicemia) AND (MicroRNAs[MeSH Terms] OR miRNAs OR MicroRNA OR miRNA). Search terms for the CNKI, EMBASE and CENTRAL with corresponding publication numbers can be found in the S1 Appendix. Language was limited in English and Chinese.

### Study selection

The titles and abstracts of studies were evaluated first. Full articles were then retrieved for potentially relevant research and reviewed for conformity with inclusion and exclusion criteria.

Criteria for inclusion: (1) all sepsis patients were confirmed by diagnosis criteria; (2) randomized or non-randomized controlled, cohort studies, clinical trials, evaluating the expression of miRNAs; (3) contained data of receiver operating characteristic (ROC) curve and the essential sample size, or the data of true positive (TP), false positive (FP), false negative (FN), and true negative (TN); (4) all studies had controls, including healthy people or infected patients; (5) full text published in English or Chinese.

Criteria for exclusion: (1) reviews, conferences articles, letters, or case reports without controls; (2) no adequate data to make a 2×2 table; (3) the total sample size of sepsis patients and controls included in the article was too small (n < 60); (4) duplicated studies.

### Data collection and assessment of study quality

Two investigators (Yue Zhang and Xiaolan Zheng) independently reviewed study eligibility of studies at the title and abstract level, using the inclusion and exclusion criteria, with third reviewer (Yifei Li) determining the divergences and report quality. All papers that met all of the criteria for inclusion would be assessed further. According to the Quality Assessment of Diagnostic Accuracy Studies 2 (QUADAS-2) list [26], two investigators (Xiaolan Zheng and Sha Lin) independently assessed all enrolled studies, and any disagreements were resolved through discussion with a third reviewer (Yifei Li). Moreover, interrater reliability for the study selection was calculated by the kappa statistic. Besides, we extracted data from the figures using Photoshop CS6 (Adobe Systems Software Ireland Ltd) using the method given in our previous report [6]. Finally, two researchers (Xiaolan Zheng and Yue Zhang) retrieved data that may be used to determine TP, TN, FP, and FN, including sensitivity, specificity, and the number of patients and controls.

### Evaluation indicators

Sensitivity, specificity, diagnostic odds ratio (DOR), and area under the summary ROC (SROC) curves values were all measured. The fraction of sepsis patients accurately identified by positive miRNA expression results was called sensitivity. Non-sepsis cases successfully recognized by negative miRNAs results were represented by specificity. DOR indicated that patients with positive results are much more likely to have sepsis than patients with negative results, and a higher DOR showed that the test had stronger discriminatory ability [27]. The area under the curve (AUC) value was analyzed as a global measurement of test performance, and the SROC curve was generated based on sensitivity and specificity. The greater the test performance, the closer the AUC value was to 1 [28].

### Publication bias

Following funnel plots and the Deeks’ test, a quantitative analysis of all the publication bias was conducted using STATA 15.1 (Stata Corporation, College Station, Texas, USA). The possibility of publication bias was highlighted by an uneven distribution of data points with a quantified result of P < 0.05 [29].

### Heterogeneity and meta-regression

Heterogeneity of pooling sensitivity and specificity was measured by x^2^ test, and the Cochran Q test was used to determine the heterogeneity of pooling DOR. When P < 0.05, statistically significant heterogeneity was present. In addition, inconsistency index (I^2^) test was used to determine the proportion of total variation between studies quantitatively. The I^2^ value ranged from 0 to 100%, with values of 25, 50, and 75% indicating low, moderate, and high heterogeneity, respectively [30]. When heterogeneity was detected, we used STATA 15.1 to do a meta-regression analysis to determine the source of heterogeneity. The correlation between possible factors and existent heterogeneities could be determined using meta-regression. When a significant difference is found, the factor should have a significant impact on the homogeneity of the included studies, with a P value of less than 0.05.

### Sensitivity analysis and subgroup analysis

The sensitivity analysis for each study was carried using STATA 15.1 to quantify the impact of specific studies on the results. Meta-Disc 1.4 was used to perform subgroup analysis and detect threshold effects in studies.

### Statistical analysis

For data processing and threshold analysis, Meta-Disc 1.4 was utilized [31]. Besides, publication bias was performed by STATA Version 15.1. Homogenous results were analyzed using the fixed effects model, while the heterogeneous (I^2^ > 50%) results utilized random effects model, and the data were presented using a forest map. Moreover, RevMan 5.4 (Cochrane, London, UK) was used to evaluate the quality of included studies based on QUADAS-2.

### Trial sequential analysis

Trial sequential analysis (TSA) was performed by the TSA 0.9.5.10 beta (Copenhagen Trial Unit) to evaluate the stability of results and the required information size (RIS) [32]. In this meta-analysis, we set the type I error rate to 5%, the type II error rate to 20%, and the statistical power to be 80%, referring to the methods of previous studies [32–34]. If the results of TSA analysis show that the cumulative Z-curve crosses the trial sequential monitoring boundary or the required information size, this indicates that the statistical evidence of this meta-analysis is reliable. Otherwise, more studies must be conducted before conclusive results can be drawn [33].

## Results

### Search results

**Fig 1:**
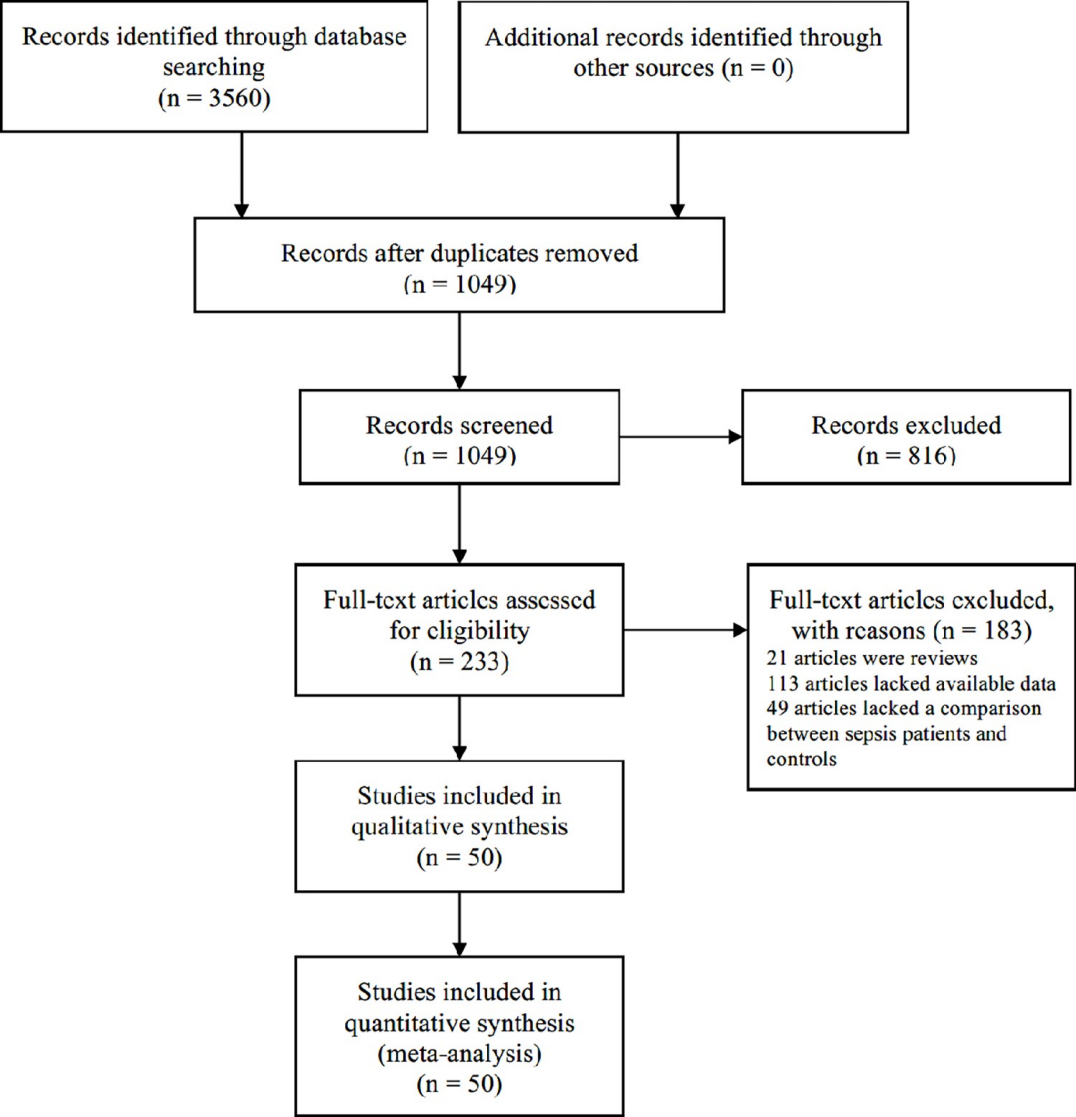
Flow diagram of the study selection process.

Initially, the search method retrieved 3560 potentially relevant studies, of which 233 studies were considered to read their whole studies after assessing titles and abstracts. However, due to article types, 21 studies were removed, and 113 studies lacked data on TP, TN, FP, and FN. Furthermore, 49 studies did not include a comparison of sepsis patients and controls. Fig 1 presents the study selection procedure. Finally, the meta-analysis comprised 50 studies [9–12, 14–23, 35–68], totaling 5225 sepsis patients and 4008 controls, and involving 48 miRNAs. Five miRNAs (miR-155-5p, miR-21, miR-223-3p, miR-146a, and miR-125a) were found to be implicated in more than two investigations. Furthermore, the age of the population was diverse. Nine studies focused on newborns less than 28 days old, four on children older than 1 month, and the remaining 37 on adults. Additionally, the sample types of 20 studies were plasma, 29 studies were serum, and one study was peripheral blood mononuclear cells (PBMC). Moreover, 46 studies from Asian (44 from China, one from India, and one from Vietnam), three from Africa (Egypt), and one from Europe (Germany). Furthermore, the 42 studies had a larger overall sample size (n ≥ 100) than the remaining eight (n < 100). Among the included articles, 44 studies followed the criteria for sepsis diagnosis were derived from sepsis 1.0 [69], sepsis 2.0 [70], sepsis 3.0 [2], while the remaining six studies did not give precise diagnostic criteria versions. In addition, 37 studies used healthy controls, whereas the remaining 13 studies used the infection controls, such as lung infection, pneumonia, and the systemic inflammatory response syndrome (SIRS). Besides, 40 studies used U6 as a qRT-PCR reference gene, eight literatures used non-U6 (miR-16-5p, SNORD61, cel-miR-39-3p, cel-miR-54), and two studies did not specify which reference gene was used. Kappa test showed the kappa value of agreement during the systematic searches was 0.87. Table 1 shows the essential characteristics of the studies that were included. The Data extracted from the included studies are shown in S2 Table.

**Table 1:** Characteristics of studies in meta-analysis.

No.	First Author	Year	Country	Population	Type of control	Golden standard	Reference genes of qRT-PCR	Specimen	Case (male/female)	Control(male/female)	Selected miRNAs
1	Sankar S	2022	India	Neonates	HC	N/R	SNORD61	Plasma	42(24/18)	42(23/19)	miR-21, miR-29a
2	Abdelaleem O	2022	Egypt	Neonates	HC	N/R	miR-16-5p	Serum	90(33/57)	90(29/61)	miR-34a-5p, miR-199a-3p
3	Wang H	2021	China	Adults	HC	Sepsis 3.0	U6	Plasma	146(87/59)	60(37/23)	miR-223-3p
4	Deng Y	2021	China	Children	HC	Sepsis 3.0	U6	Plasma	153(94/59)	60(38/22)	miR-101-3p, miR-141-3p
5	Zhang S	2021	China	Adults	HC	Sepsis 3.0	U6	Plasma	90(53/37)	92(47/45)	miR-940
6	Zhang B	2021	China	Adults	HC	Sepsis 3.0	U6	Serum	86(38/48)	85(40/45)	miR-29c-3p
7	Yao J	2021	China	Adults	HC	Sepsis 3.0	U6	Plasma	151(113/38)	15(10/5)	miR-3622b-3p, miR-519c-5p
8	Xu C	2021	China	Adults	Infection	Sepsis 3.0	U6	Serum	69(35/34)	59(30/29)	miR-21, miR-210
9	Wang Q	2021	China	Adults	HC	Sepsis 3.0	U6	Serum	80(39/41)	72(28/44)	miR-378a-3p
10	Wang D	2021	China	Neonates	Infection	N/R	U6	Serum	72(39/33)	56(32/24)	miR-1184
11	Trung N	2021	Vietnam	Adults	HC	Sepsis 3.0	miR-16-5p	Plasma	130(114/16)	82(58/24)	miR-146-3p, miR-147b, miR-155-5p, miR-223-3p
12	Sun B	2021	China	Adults	HC	Sepsis 3.0	U6	Serum	108(55/53)	101(54/47)	miR-486-5p
13	Mao Y	2021	China	Neonates	HC	N/R	U6	Serum	90(43/47)	88(37/51)	miR-455-5p
14	Liu J	2021	China	Adults	HC	Sepsis 3.0	U6	Serum	102(53/49)	105(53/52)	miR-381-3p
15	Lin X	2021	China	Neonates	Infection	N/R	U6	Serum	98(46/52)	50(22/28)	miR-141
16	Li M	2021	China	Neonates	Infection	Sepsis 2.0	U6	Serum	75(41/34)	84(47/37)	miR-129-5p
17	Zhao J	2020	China	Children	HC	Sepsis 2.0	U6	PBMC	90(55/35)	60(35/25)	miR-466
18	Yang Z	2020	China	Adults	HC	Sepsis 2.0	U6	Plasma	120(81/39)	120(68/52)	miR-103, miR-107
19	Wang J	2020	China	Adults	HC	Sepsis 3.0	N/R	Serum	82(43/39)	30(N/R)	miR-25
20	Li H	2020	China	Adults	HC	Sepsis 3.0	U6	Serum	30(19/11)	30(17/13)	miR-150, miR-107
21	Zhu X	2020	China	Adults	HC	Sepsis 2.0	U6	Plasma	120(84/36)	120(N/R)	miR-125a, miR-125b
22	Zhao D	2020	China	Adults	HC	Sepsis 3.0	U6	Plasma	150(98/52)	150(92/58)	miR-125a, miR-125b
23	Yang Y	2020	China	Adults	HC	Sepsis 3.0	U6	Plasma	102(61/41)	100(N/R)	miR-125a
24	Xu H	2020	China	Adults	HC	Sepsis 3.0	U6	Serum	103(55/48)	98(58/40)	miR-19b-3p
25	Wang H	2020	China	Adults	HC	Sepsis 2.0	U6	plasma	132(86/46)	131(76/55)	miR-146a
26	Wang H	2020	China	Adults	HC	Sepsis 3.0	U6	Serum	98(56/42)	65(38/27)	miR-451a
27	Sun B	2020	China	Adults	HC	Sepsis 1.0	U6	Serum	110(69/41)	89(58/31)	miR-328
28	Salim R	2020	Egypt	Neonates	Infection	Sepsis 2.0	U6	Serum	50(25/25)	30(14/16)	miR-101, miR-187, miR-21
29	Na L	2020	China	Adults	HC	Sepsis 3.0	U6	Plasma	219(143/76)	219(N/R)	miR-21
30	Liu W	2020	China	Adults	HC	Sepsis 3.0	U6	Plasma	196(130/66)	196(119/77)	miR-125a
31	Liu G	2020	China	Neonates	Infection	Sepsis 2.0	U6	Serum	102(53/49)	50(28/22)	miR-181a
32	Lin R	2020	China	Adults	HC	Sepsis 3.0	U6	Plasma	208(137/71)	210(134/76)	miR-126
33	Dou H	2020	China	Adults	HC	Sepsis 2.0	U6	Serum	203(117/86)	100(64/36)	miR-155-5p, miR-143
34	Chen W	2020	China	Adults	HC	Sepsis 3.0	U6	Plasma	104(62/42)	100(N/R)	miR-146b
35	Chen L	2020	China	Adults	HC	Sepsis 3.0	U6	Plasma	180(106/74)	180(117/63)	miR-146a, miR-146b
36	Li W	2019	China	Adults	HC	N/R	U6	Serum	83(52/31)	50(32/18)	miR-21
37	Zhang W	2019	China	Adults	Infection	Sepsis 3.0	U6	Plasma	44(34/10)	52(31/21)	miR-7110-5p, miR-223-3p
38	Karam R	2019	Egypt	Children	HC	Sepsis 3.0	U6	Serum	55(35/20)	60(33/27)	miR-146a
39	Guo H	2019	China	Adults	HC	Sepsis 3.0	U6	Serum	105(59/46)	100(61/39)	miR-495
40	Li J	2018	China	Adults	Infection	Sepsis 3.0	U6	Serum	41(19/22)	20(12/8)	miR-142-3p
41	Wu X	2018	China	Adults	HC	Sepsis 1.0	U6	Plasma	187(125/62)	186(112/72)	miR-223-3p
42	Rahmel T	2018	Germany	Adults	Infection	Sepsis 3.0	cel-miR-54	Serum	108(64/44)	20(9/11)	miR-122
43	Chen C	2018	China	Children	Infection	Sepsis 3.0	U6	Serum	60(36/24)	25(14/11)	miR-126-3p
44	Chao L	2018	China	Adults	HC	Sepsis 3.0	N/R	Serum	105(59/46)	35(N/R)	miR-155-5p, miR-133a-3p
45	Liu Z	2017	China	Adults	HC	Sepsis 3.0	miR-16-5p	plasma	103(47/56)	30(N/R)	miR-122a, miR-146a, miR-155a
46	Lin H	2017	China	Adults	HC	Sepsis 3.0	cel-miR-39-3p	plasma	82(65/17)	22(14/18)	miR-15b, miR-210, miR-486
47	Han Y	2016	China	Adults	Infection	Sepsis 1.0	U6	Serum	103(71/32)	95(65/30)	miR-143
48	Yao L	2015	China	Adults	Infection	Sepsis 2.0	miR-16-5p	Serum	70(36/34)	30(12/18)	miR-25
49	Wang X	2015	China	Neonates	Infection	Sepsis 2.0	U6	Serum	46(27/19)	41(25/16)	miR-15a, miR-15b, miR-16-5p, miR-223-3p
50	Deng J	2013	China	Adults	HC	Sepsis 2.0	5S rRNA	Serum	52(42/10)	23(N/R)	miR-122

HC = healthy control, miR = mircoRNA, PBMC = peripheral blood mononuclear cell, N/R = not report.

### Study quality

The quality of the included studies was assessed using the QUADAS-2, and the results showed that the risk of bias for index test was high (S1 Fig).

### Diagnostic accuracy of miRNAs

#### Total mixed miRNAs

**Fig 2:**
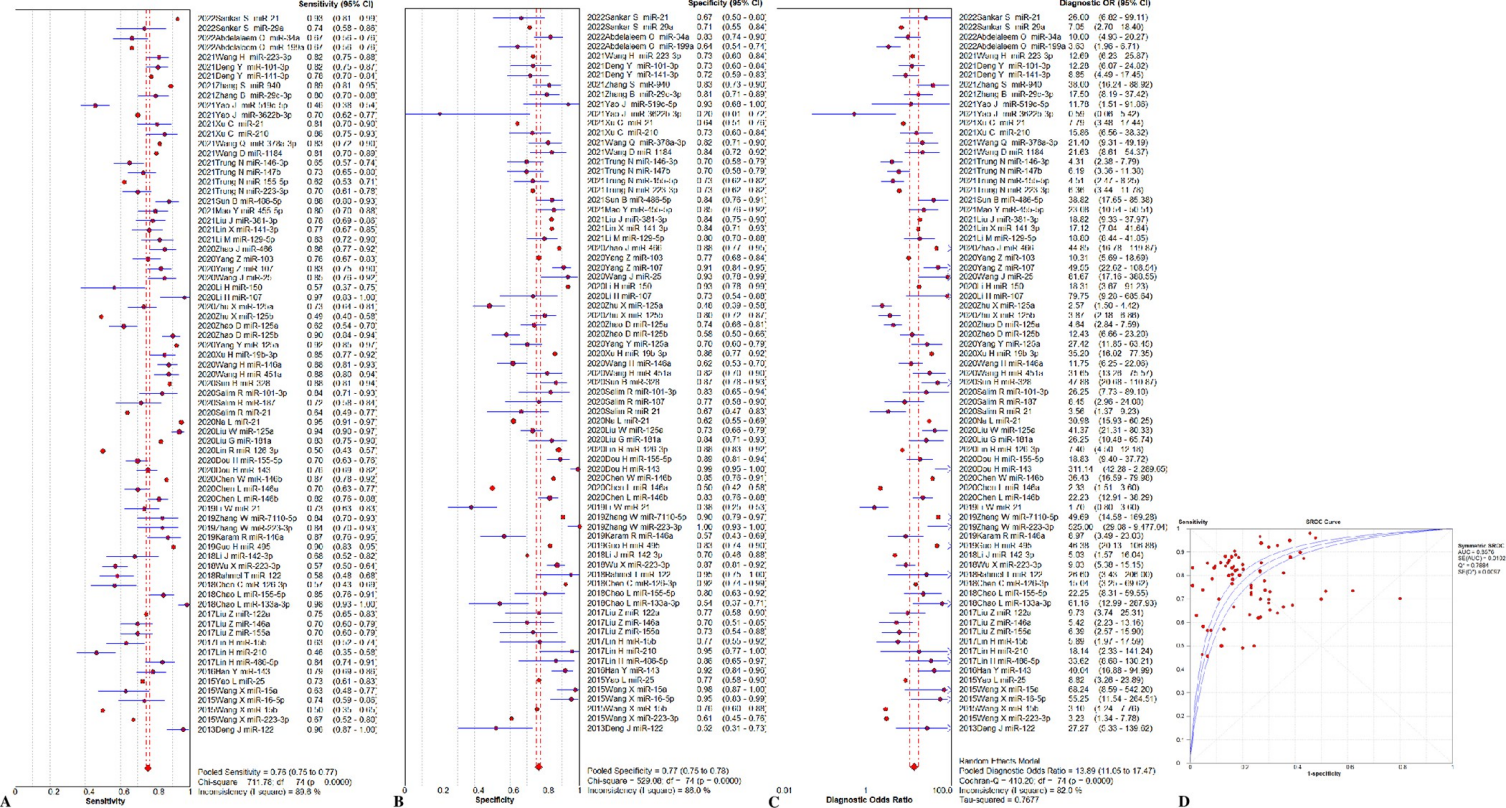
Performance of total miRNAs detection for sepsis diagnosis. (A) Pooled sensitivity. (B) Pooled specificity. (C) Overall DOR. (D) The SROCs for all datasets. CI = confidence interval, DOR = diagnostic odds ratio, miR = mircoRNA, OR = odds ratio, SROC = summary receiver operating characteristic curves value.

**Fig 3:**
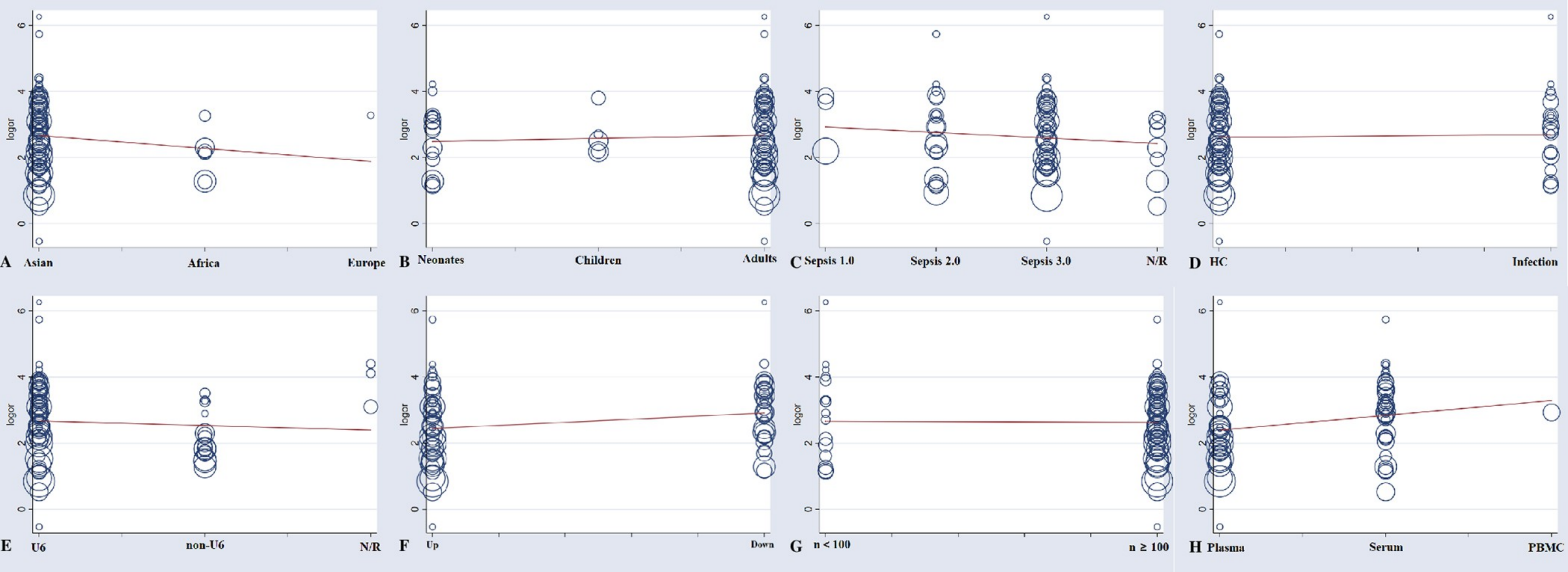
The meta-regression of the enrolled studies. (A) for the region, the meta-regression did not find it was a dramatic impact on the homogeneity of the enrolled studies, P = 0.296, t = -1.05, 95%CI (0.32, 1.42). (B) for the population, the meta-regression found no significant impact on the homogeneity of the enrolled studies, P = 0.519, t = 0.65, 95%CI (0.82, 1.47). (C) for the sepsis diagnostic criteria, the meta-regression did not detect it was a dramatic impact on the homogeneity of the enrolled studies, P = 0.331, t = -0.98, 95%CI (0.60, 1.19). (D) for the controls composition, the meta-regression did not detect it was a dramatic impact on the homogeneity of the enrolled studies, P = 0.76, t = 0.31, 95%CI (0.62, 1.91). (E) for the qRT-PCR reference genes, the meta-regression did not find it was a dramatic impact on the homogeneity of the enrolled studies, P = 0.553, t = -0.60, 95%CI (0.55, 1.38). (F) for the miRNA expression level, the meta-regression did not detect it was a dramatic impact on the homogeneity of the enrolled studies, P = 0.053, t = 1.97, 95%CI (0.99, 2.58). (G) for the total sample size, the meta-regression did not discover a significant impact on the homogeneity of the enrolled studies, P = 0.913, t = -0.11, 95%CI (0.51, 1.82). (H) for the type of samples, the meta-regression detected it was a dramatic impact on the homogeneity of the enrolled studies, P = 0.046, t = 2.03, 95%CI (1.01, 2.43).

The overall diagnostic assessment of total mixed miRNAs (TmiRs) in identifying sepsis has been summarized in Fig 2. The summary sensitivity was 0.76 (95%CI, 0.75 to 0.77), and the pooled estimation revealed significant heterogeneity (P < 0.0001, x^2^ = 711.78, I^2^ = 89.6%, Fig 2A). Meanwhile, the summary specificity was 0.77 (95%CI, 0.75 to 0.78), and the pooled estimation also showed noticeable heterogeneity (P < 0.0001, x^2^ = 529.08, I^2^ = 86.0%, Fig 2B). In addition, the pooled DOR was 13.89 (95% CI, 11.05 to 17.47) with significant heterogeneity (P < 0.0001, Cochran-Q = 410.20, I^2^ = 82.0%, Fig 2C). The calculated AUC value was 0.86 ± 0.01 (Fig 2D). Besides, the result of threshold effect analysis suggested that the Spearman correlation coefficient was 0.155 and P = 0.185, which was indicating no threshold effect related to heterogeneity existed. Next, we ran a meta-regression analysis to see what factors might be causing the heterogeneities. Type of samples, region, total sample size, sepsis diagnostic criteria, qRT-PCR reference genes, population, miRNA expression level, and controls composition were all taken into account in the meta-regression to detect the origins of heterogeneities after reviewing the baseline data and the original data producing procedure. According to the findings (Fig 3), the type of samples may be a source of heterogeneity (P = 0.046, t = 2.03, 95%CI (1.01, 2.43), Fig 3H), While the remaining seven factors are not (P > 0.05, Fig 3A–3G).

### Subgroup analysis

**Fig 4:**
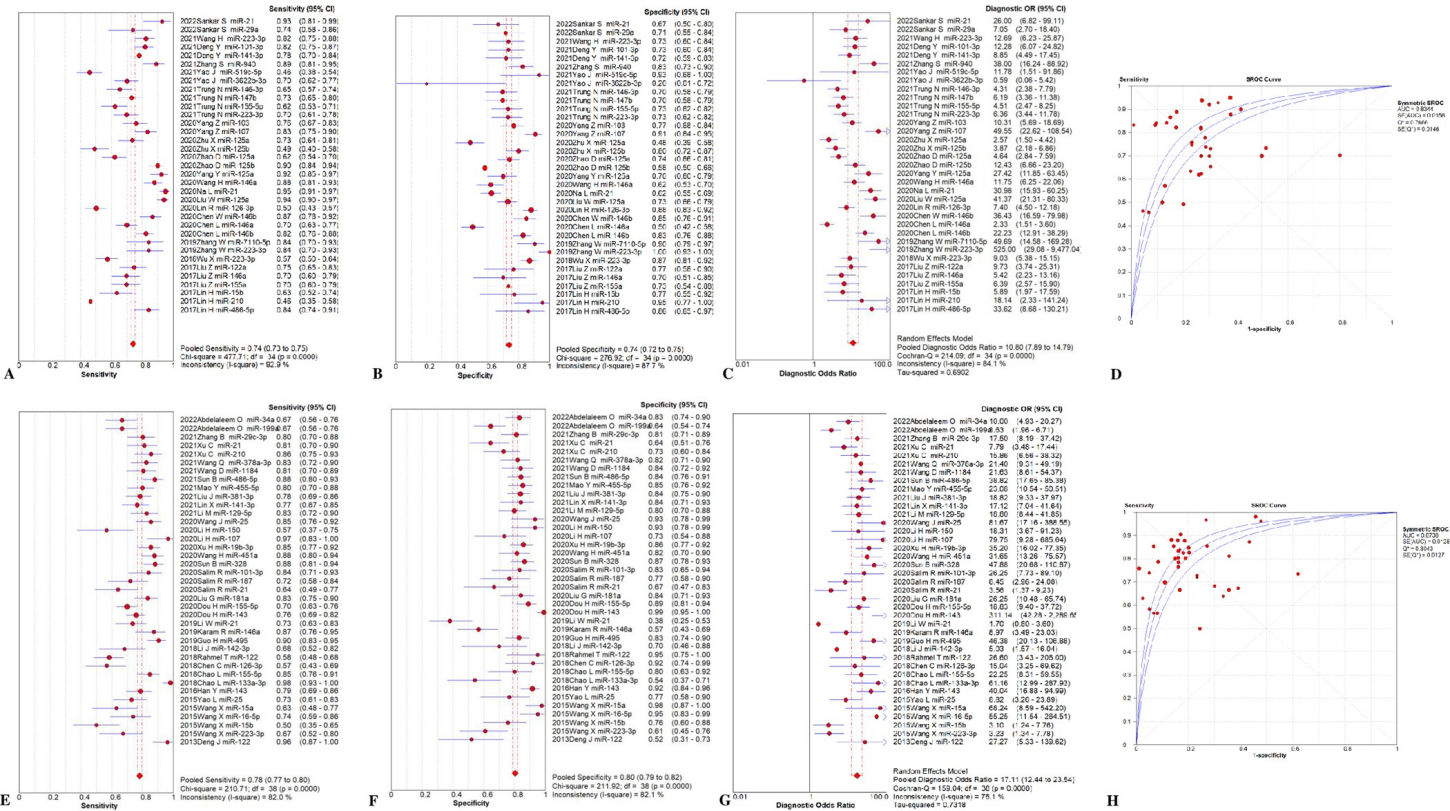
Performance of type of samples detection for sepsis diagnosis. (A) Pooled sensitivity of plasma. (B) Pooled specificity of plasma. (C) Overall DOR of plasma. (D) The SROCs of plasma. (E) Pooled sensitivity of serum. (F) Pooled specificity of serum. (G) Overall DOR of serum. (H) The SROCs of serum. CI = confidence interval, DOR = diagnostic odds ratio, miR = mircoRNA, OR = odds ratio, SROC = summary receiver operating characteristic curves value.

**Fig 5:**
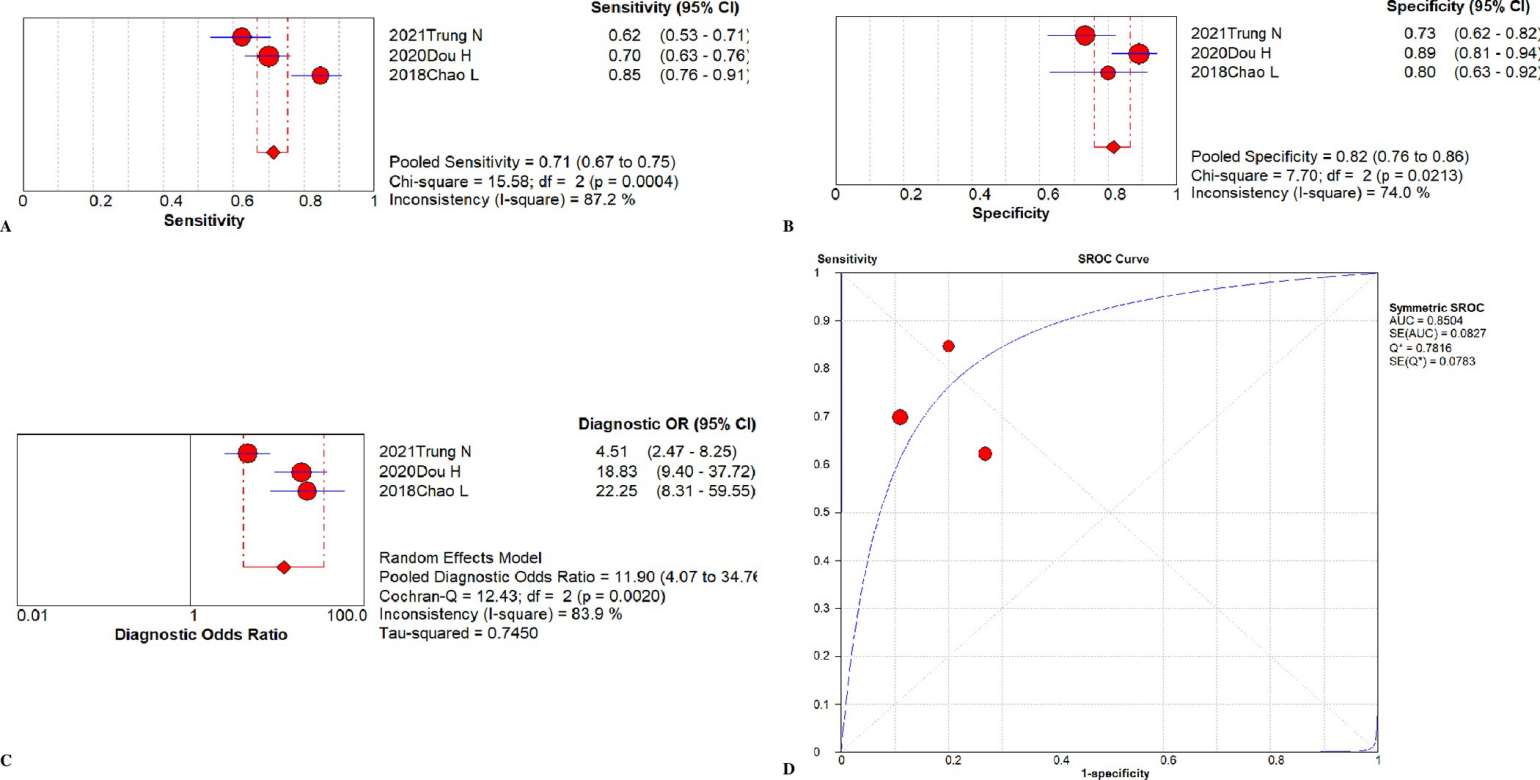
Performance of miR-155-5p for sepsis diagnosis. (A) Pooled sensitivity. (B) Pooled specificity. (C) Overall DOR. (D) The SROCs for all datasets. CI = confidence interval, DOR = diagnostic odds ratio, miR = mircoRNA, OR = odds ratio, SROC = summary receiver operating characteristic curves value.

The plasma and serum groups were subdivided to see if they were the source of heterogeneity. In addition, the analysis was carried out for five miRNAs (miR-155-5p, miR-21, miR-223-3p, miR-146a, and miR-125a), which were studied in more than two separate investigations. Prior to subgroup analysis, Meta-Disc 1.4 was used to do threshold analysis on each group’s data, and the results indicated that there was no potential for a threshold effect. Then, we analyzed each subgroup. The results were shown in Table 2, Figs 4, 5 and S2–S5 Figs.

**Table 2:** Subgroup analysis results of included studies.

	Sensitivity (95% CI)	Specificity (95% CI)	DOR (95% CI)	SROC(AUC±SE)
Type of samples				
Serum	0.78(0.77–0.80)	0.80(0.79–0.82)	17.11(12.44–23.54)	0.87±0.01
P/I^2^	< 0.0001/82%	< 0.0001/82.1%	< 0.0001/82%	-
Plasma	0.74(0.73–0.75)	0.74(0.72–0.75)	10.80(7.89–14.79)	0.83±0.02
P/I^2^	< 0.0001/92.9%	< 0.0001/87.7%	< 0.0001/84.1%	-
Type of miRNAs				
miR-155-5p	0.71(0.67–0.75)	0.82(0.76–0.86)	11.90(4.07–34.76)	0.85±0.08
P/I^2^	0.0004/87.2%	0.0213/74.0%	0.0020/83.9%	-
miR-21	0.86(0.82–0.89)	0.60(0.55–0.65)	8.05(2.45–26.40)	0.67±0.14
P/I^2^	< 0.0001/91.3%	0.0169/66.8%	< 0.0001/89.6%	-
miR-223-3p	0.70(0.66–0.74)	0.81(0.77–0.85)	8.82(4.41–17.66)	0.78±0.06
P/I^2^	< 0.0001/86.6%	< 0.0001/90.2%	0.0048/73.3%	-
miR-146a	0.77(0.73–0.81)	0.56(0.51–0.61)	5.86(2.42–14.19)	0.69±0.11
P/I^2^	0.0001/85.9%	0.0749/56.6%	0.0002/85.0%	-
miR-125a	0.81(0.77–0.84)	0.67(0.63–0.71)	10.54(2.88–38.56)	0.74±0.12
P/I^2^	< 0.0001/95.8%	< 0.0001/87.9%	< 0.0001/94.4%	-

CI = confidence interval, DOR = diagnostic odds ratio, SROC = summary receiver operating characteristic curves value, AUC = area under the curve, SE = standard error.

#### Sample type

The total diagnostic performance of the plasma group in detecting sepsis is shown in Fig 4A–4D. The pooled sensitivity was 0.74 (95%CI, 0.73 to 0.75), and the estimation showed significant heterogeneity (P < 0.0001, x^2^ = 477.71, I^2^ = 92.9%, Fig 4A). The summary specificity was 0.74 (95%CI, 0.72 to 0.75), and the pooled estimation showed high heterogeneity (P < 0.0001, x^2^ = 276.92, I^2^ = 87.7%, Fig 4B). The pooled DOR was 10.80 (95%CI, 7.89 to 14.79) with a noticeable heterogeneity (P < 0.0001, Cochran-Q = 214.09, I^2^ = 84.1%, Fig 4C). It revealed that the AUC value was 0.83 ± 0.02 (Fig 4D). Additionally, for the serum group, the overall sensitivity was 0.78 (95%CI, 0.77 to 0.80), and the pooled estimation showed significant heterogeneity (P < 0.0001, x^2^ = 210.71, I^2^ = 82.0%, Fig 4E). The summary specificity was 0.80 (95%CI, 0.79 to 0.82), and the pooled estimation showed high heterogeneity (P < 0.0001, x^2^ = 211.92, I^2^ = 82.1%, Fig 4F). Meanwhile, the pooled DOR was 17.11 (95%CI, 12.44 to 23.54) with a noticeable heterogeneity (P < 0.0001, Cochran-Q = 159.04, I^2^ = 76.1%, Fig 4G), and the AUC value was 0.87 ± 0.01 (Fig 4H).

#### miR-155-5p

Three studies [14–16] were included in examination to the overall diagnostic performance of miR-155-5p (Fig 5). The summary sensitivity was 0.71 (95%CI, 0.67 to 0.75), and the pooled estimation showed significant heterogeneity (P = 0.0004, x^2^ = 15.58, I^2^ = 87.2%, Fig 5A). Additionally, the summary specificity was 0.82 (95%CI, 0.76 to 0.86), and the pooled estimation showed moderate heterogeneity (P = 0.0213, x^2^ = 7.70, I^2^ = 74.0%, Fig 5B). The pooled DOR was 11.90 (95%CI, 4.07 to 34.76) with a noticeable heterogeneity (P = 0.0020, Cochran-Q = 12.43, I^2^ = 83.9%, Fig 5C). It revealed that the AUC was 0.85 ± 0.08 (Fig 5D).

#### miR-21

Five studies [9, 10, 17, 53, 59] were included to evaluate the overall diagnostic performance of miR-21 (S2 Fig). The summary sensitivity was 0.86 (95%CI, 0.82 to 0.89), and the specificity was 0.60 (95%CI, 0.55 to 0.65). Both pooled estimations showed significant heterogeneity (sensitivity: P < 0.0001, x^2^ = 45.85, I^2^ = 91.3%; specificity: P = 0.0169, x^2^ = 12.06, I^2^ = 66.8%, S2A and S2B Fig). The pooled DOR was 8.05 (95%CI, 2.45 to 26.40), with a noticeable heterogeneity (P < 0.0001, Cochran-Q = 38.52, I^2^ = 89.6%, S2C Fig). Besides, the AUC value was 0.67 ± 0.14 (S2D Fig).

#### miR-223-3p

Five studies [12, 14, 40, 58, 61] were included to analysis the overall diagnostic performance of miR-223-3p (S3 Fig). The summary sensitivity for the diagnostic performance of miR-223-3p was 0.70 (95%CI, 0.66 to 0.74), and the specificity was 0.81 (95%CI, 0.77 to 0.85). High heterogeneity showed in the results (sensitivity: P < 0.0001, x^2^ = 29.88, I^2^ = 86.6%; specificity: P < 0.0001, x^2^ = 40.71, I^2^ = 90.2%, S3A,and S3B Fig). The pooled DOR was 8.82 (95%CI, 4.41 to 17.66), with moderate heterogeneity (P = 0.0048, Cochran-Q = 14.97, I^2^ = 73.3%, S3C Fig), and the AUC value was 0.78 ± 0.06 (S3D Fig).

#### miR-146a

Four studies [18–20, 64] were included to assess the overall diagnostic performance of miR-146a (S4 Fig). The summary sensitivity was 0.77 (95%CI, 0.73 to 0.81), and the specificity was 0.56 (95%CI, 0.51 to 0.61). Both pooled estimations showed significant heterogeneity (sensitivity: P = 0.0001, x^2^ = 21.33, I^2^ = 85.9%; specificity: P = 0.0749, x^2^ = 6.91, I^2^ = 56.6%, S4A and S4B Fig). The pooled DOR was 5.86 (95%CI, 2.42 to 14.19), with high heterogeneity (P = 0.0002, Cochran-Q = 20.01, I^2^ = 85.0%, S4C Fig). Besides, the AUC value was 0.69 ± 0.11 (S4D Fig).

#### miR-125a

Four studies [21–23, 54] were included to analysis the overall diagnostic performance of miR-125a (S5 Fig). The summary sensitivity was 0.81 (95%CI, 0.77 to 0.84), and the specificity was 0.67 (95%CI, 0.63 to 0.71). High heterogeneity showed in the results (sensitivity: P < 0.0001, x^2^ = 70.70, I^2^ = 95.8%; specificity: P < 0.0001, x^2^ = 24.78, I^2^ = 87.9%, S5A and S5B Fig). The pooled DOR was 10.54 (95%CI, 2.88 to 38.56), with a noticeable heterogeneity (P < 0.0001, Cochran-Q = 53.83, I^2^ = 94.4%, S5C Fig), and the AUC value was 0.74 ± 0.12 (S5D Fig).

### Sensitivity analysis and publication bias

**Fig 6:**
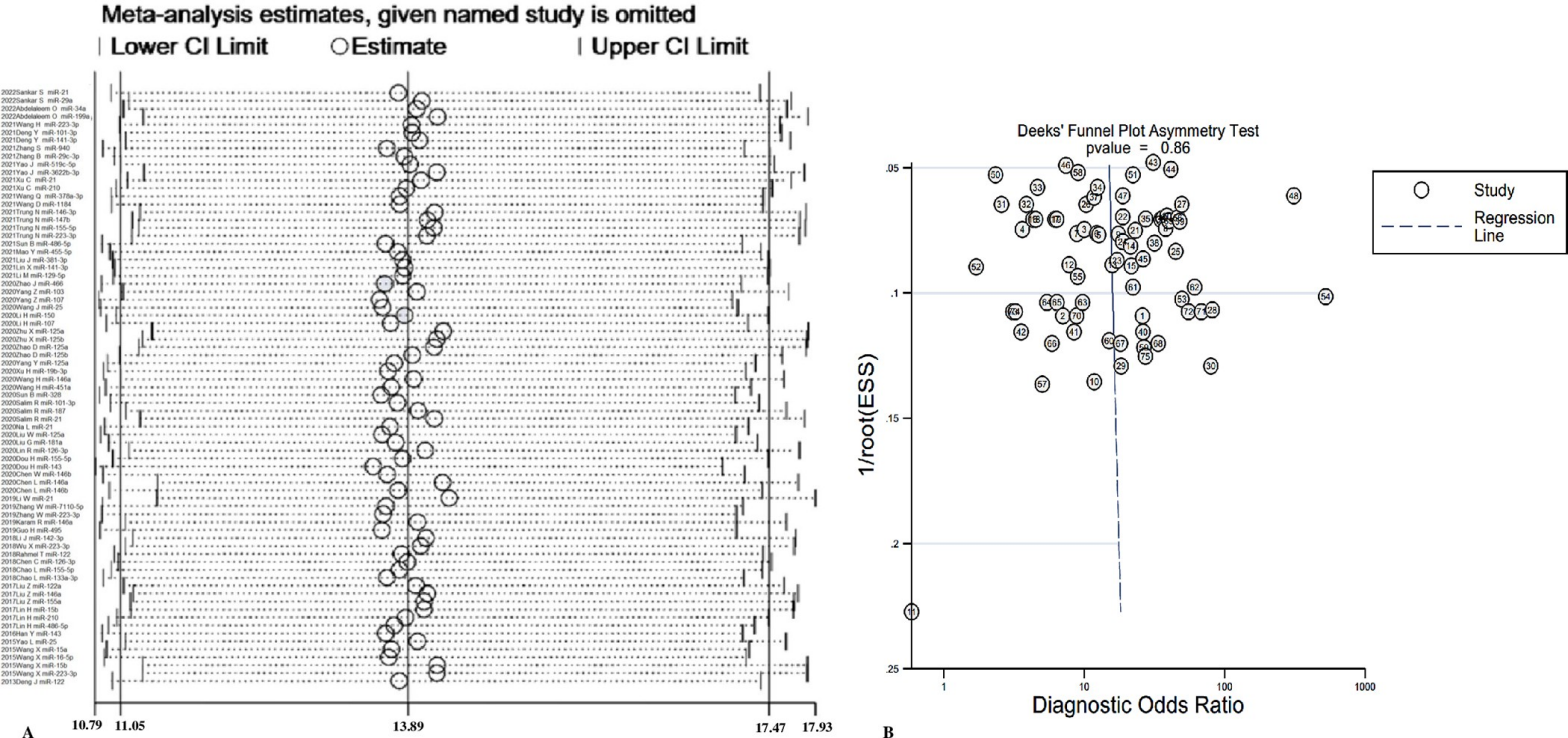
Sensitivity analysis and publication bias of the individual trials on the results TmiRs. (A) Sensitivity analysis for the result of TmiRs, (B) Funnel plot for the assessment of potential publication bias. DOR = diagnostic odds ratio, ESS = effective sample size, miR = mircoRNA, TmiRs = total mixed miRNAs.

We found no significant influence from any of the studies, and STATA 15.1 corroborated the TmiRs results (Fig 6A). Furthermore, funnel plots were utilized to assess publication bias in the included papers, and no significant publication biases were found (P = 0.859, 95% CI, -10.58 to 12.66) (Fig 6B).

### Trial sequential analysis

TSA results demonstrated that the cumulative Z-score of TmiR, miR-155-5p, miR-223-3p, and miR-125a crossed its monitoring boundaries and reliable conclusions had been drawn. But the miR-21 and miR-146a did not reach the required sample size (S6 Fig).

## Discussion

Many studies have been conducted to see if miRNAs may be utilized as biomarkers for sepsis since their emergence about a decade ago. In this meta-analysis, we enrolled 50 studies totaling 5225 sepsis patients and 4008 controls, involving 48 miRNAs. Finally, we discovered that TmiRs had a combined AUC of 0.86, with 0.76 pooled sensitivity and 0.77 specificity, indicating that miRNAs had a moderate diagnostic accuracy as a diagnostic biomarker in discriminating sepsis. In addition, we examined individual miRNAs in the overall miRNA library and discovered that miR-155-5p, miR-21, miR-223-3p, miR-146a, and miR-125a were the most often used in recent studies. We discovered that among all miRNAs, miR-155-5p had the highest AUC of SROC: pooled sensitivity, 0.71 (95%CI, 0.67 to 0.75); pooled specificity, 0.82 (95%CI, 0.76 to 0.86); and SROC, 0.85; indicating that miR-155-5p had reasonable diagnostic accuracy in identifying sepsis. To our knowledge, this is the first meta-analysis that focused on the accuracy of miR-155-5p, miR-21, miR-146a, and miR-125a in detecting sepsis. This meta-analysis was crucial in determining the potential for miRNAs to be utilized to diagnose sepsis.

It is worth noting that not every study provides cut-off value of miRNA. In addition, possibly due to factors such as qRT-PCR reference genes and the source of samples, the same miRNA was used in different papers with different cut-off values. Moreover, selecting the test cut-off to optimize sensitivity and/or specificity may lead to overestimation of test performance. These reasons may have contributed to the high risk of index test by QUADAS-2. Therefore, we did threshold analysis before each analysis and found no threshold effect, indicating that there was no threshold effect related to heterogeneity. Besides, as some pooled results showed large heterogeneity, type of samples, region, total sample size, sepsis diagnostic criteria, qRT-PCR reference genes, population, miRNA expression level, and controls composition were all analysis in the meta-regression to detect the origins of heterogeneity. Finally, the specimen type was discovered to be a source of heterogeneity, and the results of the following subgroup analysis revealed that serum miRNAs might be employed as sepsis diagnostic biomarkers compared to plasma (SROC: 0.87, 0.83, respectively).

Some studies [40, 44, 46] in our included literature focused on the ability of miRNA, PCT, CRP, and other markers to jointly diagnose sepsis. It showed that the diagnostic accuracy of combined markers was higher than that of single marker. Even so, we did not undertake a combined analysis of miRNAs and other indicators due to a lack of research and the various types of miRNA combinations discovered. Nonetheless, this approach to integrated diagnosis has a lot of promise. Previous studies [13, 71] have also indicated the importance of combined diagnosis in sepsis, however additional clinical investigations are needed in the future to clarify the signs of combined diagnosis.

In 2020, Shen et al. [13] conducted a meta-analysis in 2020 with a total of 22 studies, comprising 2210 sepsis and 1502 controls, to validate the diagnostic accuracy of miRNAs for sepsis, and discovered that miR-223-3p may be employed as a sepsis indicator. However, we discovered that miR-223-3p had worse diagnostic accuracy than miR-155-5p in our research with a wider literature and patient group (SROC: 0.78, 0.85, respectively). Nonetheless, the diagnostic accuracy of miR-223-3p was higher than that of miR-21, miR-146a, and miR-125a (SROC: 0.67, 0.69, and 0.74, respectively), which have received more attention in recent years. Therefore, the importance of miR-223-5p in sepsis detection cannot be overlooked. More crucially, the following are the benefits of our meta-analysis: First, this meta-analysis included more than twice as many studies as the previous one. Second, we tested the diagnostic accuracy of five miRNAs that may be used as sepsis biomarkers, and discovered that miR-155-5p was a crucial miRNA among the total miRNAs. Third, the subgroup analyses discovered that serum miRNAs had the best diagnostic accuracy. Moreover, we performed the TSA to evaluate the stability of results and the RIS, which showed the sample size included in the meta-analysis of TmiR, miR-155-5p, miR-223-3p, and miR-125a was sufficient and significant associations were observed.

There are also several limitations of this meta-analysis that must be addressed. First, our meta-analysis included 48 miRNA markers, with only 5 miRNA markers appearing in three to five of the publications included. Among them, the miR-155-5p showed the highest diagnostic ability. However, only three studies included to evaluate the diagnostic accuracy of miR-155-5p, which might lead to bias in meta-analysis results. Second, cross-comparisons between studies conducted by different laboratories are limited due to the lack of traditional methods for accurate and absolute quantification of miRNAs, as well as the inconsistent reference genes of qRT-PCR and sample types used by various laboratories, resulting in unconvincing results for the included studies.

## Conclusions

In conclusion, our meta-analysis demonstrated that miRNAs, specifically miR-155-5p, could be valuable biomarkers for detecting sepsis. For diagnostic purposes, a clinical serum specimen is also required. To assess the usefulness of miRNA in the detection of sepsis in the future, more well-designed and harmonized clinical trials will be required.

## Supporting information

S1 Table PRISMA checklist. (DOC)

S2 Table Data extracted from the included studies. (DOCX)

S1 Appendix Search strategies for EMBASE, the Cochrane Central Register of Controlled Trials, and China National Knowledge Infrastructure. (DOCX)

S1 Fig Quality assessment of the included studies according to QUADAS-2. (A) Methodological quality graph; (B) Methodological quality summary.
(PDF)

S2 Fig Performance of miR-21 detection for sepsis diagnosis. (A) Pooled sensitivity. (B) Pooled specificity. (C) Overall DOR. (D) The SROCs for all datasets.
(PDF)

S3 Fig Performance of miR-223-3p detection for sepsis diagnosis. (A) Pooled sensitivity. (B) Pooled specificity. (C) Overall DOR. (D) The SROCs for all datasets.
(PDF)

S4 Fig Performance of miR-146a detection for sepsis diagnosis. (A) Pooled sensitivity. (B) Pooled specificity. (C) Overall DOR. (D) The SROCs for all datasets.
(PDF)

S5 Fig Performance of miR-125a detection for sepsis diagnosis. (A) Pooled sensitivity. (B) Pooled specificity. (C) Overall DOR. (D) The SROCs for all datasets.
(PDF)

S6 Fig TSA plot of miRNAs for sepsis diagnosis. (A) TSA plot of TmiR for sepsis diagnosis. (B) TSA plot of miR-155-5p for sepsis diagnosis. (C) TSA plot of miR-21 for sepsis diagnosis. (D) TSA plot of miR-223-3p for sepsis diagnosis. (E) TSA plot of miR-146a for sepsis diagnosis. (F) TSA plot of miR-125a for sepsis diagnosis.
(PDF)

## Abbreviations

CRP: C-reactive protein
PCT: procalcitonin
miRNAs: microRNAs
CI: confidence interval
SROC: summary receiver operating characteristic curves value
AUC: area under the curve
PRISMA: Preferred Reporting Items for Systematic Reviews and Meta-Analyses
CNKI: China National Knowledge Infrastructure
CENTRAL: Cochrane Central Register of Controlled Trials
ROC: receiver operating characteristic
TP: true positive false positive
FP: false positive
FN: false negative
TN: true negative
QUADAS: Quality Assessment of Diagnostic Accuracy Studies
DOR: diagnostic odds ratio
I^2^: inconsistency index
TSA: trial sequential analysis
RIS: required information size
PBMC: peripheral blood mononuclear cells
SIRS: systemic inflammatory response syndrome
TmiRs: total mixed miRNAs
OR: odds ratio
ESS: effective sample size
HC: healthy control
N/R: not report
SE: standard error
