# Supplementary material for: Diagnostic significance of microRNAs in sepsis
Source: PLoS One. 2023 Feb 22;18(2):e0279726. doi: 10.1371/journal.pone.0279726 (PMC9946237; doi:10.1371/journal.pone.0279726)
Supplement: S1 Table — (DOC) [file pone.0279726.s001.doc]

| **Section/topic** | **#** | **Checklist item** | **Reported on page #** |
| --- | --- | --- | --- |
| **TITLE** | | |  |
| Title | 1 | Diagnostic significance of miRNAs in sepsis | 1 |
| ABSTRACT | | |  |
| Structured summary | 2 | Background  Sepsis is a life-threatening condition that induce tens of million death each year, yet early diagnosis remains a formidable challenge. Many studies have focused on the diagnostic accuracy of microRNAs (miRNAs) for sepsis in recent years, particularly miR-155-5p, miR-21, miR-223-3p, miR-146a, and miR-125a. Thus, we conducted this meta-analysis to explore if miRNAs may be used as a biomarker for sepsis detection.  Methods  We searched PubMed, the Cochrane Central Register of Controlled Trials, EMBASE, and China National Knowledge Infrastructure through May 12, 2022. This meta-analysis was conducted using Meta-disc 1.4 and STATA 15.1 in a fixed/random-effect model.  Results  The analysis included a total of 50 relevant studies. The overall performance of total miRNAs detection was: pooled sensitivity, 0.76 (95% confidence interval [CI], 0.75 to 0.77); pooled specificity, 0.77 (95%CI, 0.75 to 0.78); and area under the summary receiver operating characteristic curves value (SROC), 0.8576. The subgroup analysis suggested that detection in miR-155-5p group had the highest AUC of SROC among all miRNAs: pooled sensitivity, 0.71 (95%CI, 0.67 to 0.75); pooled specificity, 0.82 (95%CI, 0.76 to 0.86); and SROC, 0.8504. MiR-21, miR-223-3p, miR-146a, and miR-125a had SROC values of 0.6691, 0.7847, 0.6946, and 0.7430, respectively. The specimen type was found to be a source of heterogeneity in the meta-regression study. The SROC of serum was higher than that of plasma (0.8738 and 0.8344, respectively).  Conclusions  Our meta-analysis revealed that miRNAs, specifically miR-155-5p, could be useful biomarkers for detecting sepsis. A clinical serum specimen is also indicated for diagnostic purposes. | 1-2 |
| INTRODUCTION | | |  |
| Rationale | 3 | Sepsis is a life-threatening disease that induce about 11 million death each year. The main cause of sepsis is the maladjustment of the host's response to infection. Sepsis is treatable and timely implementation of targeted interventions can improve outcomes. Meanwhile, delayed diagnosis is associated with increased mortality. Therefore, early diagnosis of sepsis is the key to improve the survival rate. Nevertheless, sepsis is a heterogeneous syndrome and the diagnosis of sepsis detection is mainly according to the site of infection, etiology, onset time, and the patient’s profile. However, traditional screening methods and biomarkers such as CRP and PCT lack specificity, which leads to the early diagnosis of sepsis is still a formidable challenge. Therefore, it is important to find novel and reliable biomarkers for early diagnosis of sepsis.  MicroRNAs (miRNAs) are a type of small noncoding RNA with an average length of 18-25 nucleotides. Previous research has discovered that circulating miRNAs can be used as biomarkers to detect various diseases. Vasilescu et al. were the fist to discover that plasma miR-150 was a potential biomarker for sepsis. Numerous studies have since confirmed the significance of miRNAs in sepsis. Several miRNAs have been mentioned in several studies with varying diagnostic effectiveness. Shen et al. conducted a meta-analysis in 2020 to validate the diagnostic accuracy of miRNAs for sepsis, and found that miR-223-3p might be used as a sepsis indicator. In recent years, however, a growing number of research have focused on the diagnostic accuracy of miRNAs for sepsis, particularly miR-155-5p, miR-21, miR-146a, and miR-125a, In recent years, however, a growing number of research have focused on the diagnostic accuracy of miRNAs for sepsis, particularly miR-155-5p[21-23], miR-21, miR-146a[24-26], and miR-125a, but the results have been inconsistent. Thus, we collected all published case-control articles to gather evidence on the diagnostic accuracy of miRNAs for sepsis. | 2 |
| Objectives | 4 | We collected all published case-control studies to gather evidence on how the diagnostic performance of miRNAs distinguished sepsis. | 2 |
| METHODS | | |  |
| Protocol and registration | 5 | This analysis was performed by a predetermined protocol following the recommendations of Deeks. The data collection and reporting accorded with the Preferred Reporting Items for Systematic Reviews and Meta-Analyses (PRISMA) Statement. The ethical approval was not necessarily due to it is systematic literature research. | 3 |
| Eligibility criteria | 6 | Criteria for inclusion: (1) all sepsis patients were confirmed by diagnosis criteria; (2) randomized or non-randomized controlled, cohort studies, clinical trials, evaluating the expression of miRNAs; (3) contained data of receiver operating characteristic (ROC) curve and the essential sample size, or the data of true positive (TP), false positive (FP), false negative (FN), and true negative (TN); (4) all studies had controls, including healthy people or infected patients; (5) full text published in English or Chinese.  Criteria for Exclusion: (1) reviews, conferences articles, letters, or case reports without controls; (2) no adequate data to make a 2×2 table; (3) the total sample size of sepsis patients and controls included in the article was too small (n < 60); (4) duplicated studies | 4 |
| Information sources | 7 | To find relevant studies, we searched PubMed, China National Knowledge Infrastructure (CNKI), EMBASE, and the Cochrane Central Register of Controlled Trials (CENTRAL) databases until May 12, 2022. | 3 |
| Search | 8 | Keyword search terms were (‘sepsis’ OR ‘pyemia’ OR ‘septicemia’) AND (‘MicroRNAs’ OR ‘miRNAs’ OR ‘MicroRNAs’ OR ‘miRNA’). PubMed database was searched as follows: (Sepsis[MeSH Terms] OR pyemia OR septicemia) AND (MicroRNAs[MeSH Terms] OR miRNAs OR MicroRNA OR miRNA). Search terms for the CNKI, EMBASE and CENTRAL with corresponding publication numbers can be found in the S Appendix. Language was limited in English and Chinese. | 3 |
| Study selection | 9 | Reports were preliminarily screened by title and abstract and when initially selected by the systematic search. Potentially relevant studies were then retrieved by full manuscripts and assessed for compliance with inclusion and exclusion criteria. | 3 |
| Data collection process | 10 | Two investigators (Yue Zhang and Xiaolan Zheng) independently reviewed study eligibility of studies at the title and abstract level, using the inclusion and exclusion criteria, with third reviewer (Yifei Li) determining the divergences and report quality. All papers that met all of the criteria for inclusion would be assessed further. According to the 14-item Quality Assessment of Diagnostic Accuracy Studies (QUADAS) list, two investigators (Xiaolan Zheng and Sha Lin) independently assessed all enrolled reports, and any disagreements were resolved through discussion with a third reviewer (Yifei Li). Besides, we extracted data from the figures using Photoshop CS6 (Adobe Systems Software Ireland Ltd) using the method given in our previous report. Finally, two researchers (Xiaolan Zheng and Yue Zhang) retrieved data that may be used to determine TP, TN, FP, and FN, including sensitivity, specificity, and the number of patients and controls. | 3-4 |
| Data items | 11 | the data of true positive, false positive, false negative, and true negative; or the data of the receiver operating characteristic (ROC) curve, and essential sample size | 4 |
| Risk of bias in individual studies | 12 | We used Stata statistical software (STATA, version 15.1) to obtain a quantitative analysis of all the publication bias according to funnel plots and the Deek’s test. An asymmetric distribution of data points in the funnel plot with a quantified result of P<.05 indicated the presence of potential publication bias | 4 |
| Summary measures | 13 | The following indicators of different types of miRNAs were measured: sensitivity, specificity, diagnostic odds ratio (DOR), and area under the summary receiver operating characteristic curves value (SROC). | 4 |
| Synthesis of results | 14 | Sensitivity, specificity, diagnostic odds ratio (DOR), and area under the summary receiver operating characteristic curves value (SROC). | 5 |

Page 1 of 2

| **Section/topic** | **#** | **Checklist item** | **Reported on page #** |
| --- | --- | --- | --- |
| Risk of bias across studies | 15 | We used Stata statistical software (STATA, version 15.1) to obtain a quantitative analysis of all the publication bias according to funnel plots and the Deek’s test. An asymmetric distribution of data points in the funnel plot with a quantified result of P<.05 indicated the presence of potential publication bias | 5 |
| Additional analyses | 16 | We carried out the meta-regression analysis using STATA 15.1 to detect where the potential factor for heterogeneity origin from. Sensitivity analysis was conducted for every study to determine the influence of individual trials on the results, using STATA 15.1 for meta-analysis fixed/random-effects estimates. Meta-Disc 1.4 was used to detect threshold effects in studies and conduct subgroup analysis. | 5 |
| RESULTS | | |  |
| Study selection | 17 | Initially, the search method retrieved 3560 potentially relevant papers, of which 233 articles were considered to read their whole articles after assessing titles and abstracts. However, due to article types, 21 papers were removed, and 113 papers lacked data on TP, TN, FP, and FN. Furthermore, 49 articles did not include a comparison of sepsis patients and controls. Finally, the meta-analysis comprised 50 studies, totaling 5225 sepsis patients and 4008 controls, and involving 48 miRNAs. | 5 |
| Study characteristics | 18 | Finally, the meta-analysis comprised 50 studies, totaling 5225 sepsis patients and 4008 controls, and involving 48 miRNAs. Five miRNAs (miR-155-5p, miR-21, miR-223-3p, miR-146a, and miR-125a) were found to be implicated in more than two investigations. Furthermore, the age of the population was diverse. Nine studies focused on newborns less than 28 days old, four on children older than 1 month, and the remaining 37 on adults. Additionally, the sample types of 20 studies were plasma, 29 studies were serum, and one report was peripheral blood mononuclear cells (PBMC). Moreover, 46 reports from Asian (44 from China, one from India, and one from Vietnam), three from Africa (Egypt), and one from Europe (Germany). Furthermore, the 42 studies had a larger overall sample size (n ≥ 100) than the remaining eight (n < 100). Among the included articles, 44 reports followed the criteria for sepsis diagnosis were derived from sepsis 1.0, sepsis 2.0, sepsis 3.0, while the remaining six articles did not give precise diagnostic criteria versions. In addition, 37 studies used healthy controls, whereas the remaining 13 reports used the infection controls, such as lung infection, pneumonia, and the systemic inflammatory response syndrome (SIRS). Besides, 40 articles used U6 as a qRT-PCR reference gene, eight literatures used non-U6 (miR-16-5p, SNORD61, cel-miR-39-3p, cel-miR-54), and two studies did not specify which reference gene was used. Table 1 shows the essential characteristics of the articles that were included. | 5-6 |
| Risk of bias within studies | 19 | We found no significant influence from any of the studies, and STATA 15.1 corroborated the TmiRs results (Fig 6A). Furthermore, funnel plots were utilized to assess publication bias in the included papers, and no significant publication biases were found (P = 0.859, 95% CI, -10.58 to 12.66) | 9 |
| Results of individual studies | 20 | we examined individual miRNAs in the overall miRNA library and discovered that miR-155-5p, miR-21, miR-223-3p, miR-146a, and miR-125a were the most often used in recent studies. We discovered that among all miRNAs, miR-155-5p had the highest AUC of SROC: pooled sensitivity, 0.71 (95%CI, 0.67 to 0.75); pooled specificity, 0.82 (95%CI, 0.76 to 0.86); and SROC, 0.8504; indicating that miR-155-5p had reasonable diagnostic accuracy in identifying sepsis. To our knowledge, this is the first meta-analysis that focused on the accuracy of miR155-5p, miR-21, miR-146a, and miR-125a in detecting sepsis. This meta-analysis was an important study to evaluate the potential for miRNAs to be used in the diagnosis of sepsis. | 7-9 |
| Synthesis of results | 21 | The overall diagnostic assessment of total mixed miRNA (TmiR) in identifying sepsis has been summarized in Fig 2. The summary sensitivity was 0.76 (95%CI, 0.75 to 0.77), and the pooled estimation revealed significant heterogeneity (P < 0.0001, x2 = 711.78, I2 = 89.6%, Fig 2A). Meanwhile, the summary specificity was 0.77 (95%CI, 0.75 to 0.78), and the pooled estimation also showed noticeable heterogeneity (P < 0.0001, x2 = 529.08, I2 = 86.0%, Fig 2B). In addition, the pooled DOR was 13.89 (95% CI, 11.05 to 17.47) with significant heterogeneity (P < 0.0001, Cochran-Q = 410.20, I2 = 82.0%, Fig 2C). The calculated AUC value was 0.8576 ± 0.0102 (Fig 2D). | 7-9 |
| Risk of bias across studies | 22 | We found no significant influence from any of the studies, and STATA 15.1 corroborated the TmiRs results (Fig 6A). Furthermore, funnel plots were utilized to assess publication bias in the included papers, and no significant publication biases were found (P = 0.859, 95% CI, -10.58 to 12.66) | 9 |
| Additional analysis | 23 | As some pooled results showed large heterogeneities, type of samples, region, total sample size, sepsis diagnostic criteria, qRT-PCR reference genes, population, miRNA expression level, and controls composition were all analysis in the meta-regression to detect the origins of heterogeneities. Finally, the specimen type was discovered to be a source of heterogeneity, and the results of the following subgroup analysis revealed that serum miRNAs might be employed as sepsis diagnostic biomarkers compared to plasma (SROC: 0.8738, 0.8344, respectively). | 6-7 |
| **DISCUSSION** | | |  |
| Summary of evidence | 24 | In this meta-analysis, we enrolled 50 studies totaling 5225 sepsis patients and 4008 controls, involving 48 miRNAs. Finally, we discovered that TmiRs had a combined AUC of 0.8576, with 76% pooled sensitivity and 77% specificity, indicating that miRNAs had a moderate diagnostic accuracy as a diagnostic biomarker in discriminating sepsis. In addition, we examined individual miRNAs in the overall miRNA library and discovered that miR-155-5p, miR-21, miR-223-3p, miR-146a, and miR-125a were the most often used in recent studies. We discovered that among all miRNAs, miR-155-5p had the highest AUC of SROC: pooled sensitivity, 0.71 (95%CI, 0.67 to 0.75); pooled specificity, 0.82 (95%CI, 0.76 to 0.86); and SROC, 0.8504; indicating that miR-155-5p had reasonable diagnostic accuracy in identifying sepsis. To our knowledge, this is the first meta-analysis that focused on the accuracy of miR155-5p, miR-21, miR-146a, and miR-125a in detecting sepsis. | 9 |
| Limitations | 25 | There are also several limitations of this meta-analysis that must be addressed. First, our meta-analysis included 48 miRNA markers, with only 5 miRNA markers appearing in three to five of the publications included. Only three of them looked at the diagnostic ability of miR-155-5p, which could lead to skewed meta-analysis results. Second, cross-comparisons between studies conducted by different laboratories are limited due to the lack of traditional methods for accurate and absolute quantification of miRNAs, as well as the inconsistent reference genes of qRT-PCR and sample types used by various laboratories, resulting in unconvincing results for the included studies. | 10 |
| Conclusions | 26 | In conclusion, our meta-analysis demonstrated that miRNAs, specifically miR-155-5p, could be valuable biomarkers for detecting sepsis. For diagnostic purposes, a clinical serum specimen is also required. To assess the usefulness of miRNA in the detection of sepsis in the future, more well-designed and harmonized clinical trials will be required. | 11 |
| **FUNDING** | | |  |
| Funding | 27 | None. |  |

*From:*  Moher D, Liberati A, Tetzlaff J, Altman DG, The PRISMA Group (2009). Preferred Reporting Items for Systematic Reviews and Meta-Analyses: The PRISMA Statement. PLoS Med 6(7): e1000097. doi:10.1371/journal.pmed1000097

For more information, visit: **www.prisma-statement.org**.

Page 2 of 2
