# Supplementary material for: Diagnostic significance of microRNAs in sepsis
Source: PLoS One. 2023 Feb 22;18(2):e0279726. doi: 10.1371/journal.pone.0279726 (PMC9946237; doi:10.1371/journal.pone.0279726)
Supplement: S2 Table — (DOCX) [file pone.0279726.s002.docx]

| **S Table 3** Data extracted from the included studies. | | | | | |
| --- | --- | --- | --- | --- | --- |
| Study ID | TP | FP | FN | TN | Cut-off |
| 2022Sankar S miR-21 | 39 | 14 | 3 | 28 | 1.12 |
| 2022Sankar S miR-29a | 31 | 12 | 11 | 30 | 2.49 |
| 2022Abdelaleem O miR-34a | 60 | 15 | 30 | 75 | N/R |
| 2022Abdelaleem O miR-199a | 60 | 32 | 30 | 58 | N/R |
| 2021Wang H miR-223-3p | 120 | 16 | 26 | 44 | 1.08 |
| 2021Deng Y miR-101-3p | 125 | 16 | 28 | 44 | 1.18 |
| 2021Deng Y miR-141-3p | 119 | 17 | 34 | 43 | 0.72 |
| 2021Zhang S miR-940 | 80 | 16 | 10 | 76 | N/R |
| 2021Zhang B miR-29c-3p | 69 | 16 | 17 | 69 | N/R |
| 2021Yao J miR-519c-5p | 69 | 1 | 82 | 14 | 2/2.5 |
| 2021Yao J miR-3622b-3p | 106 | 4 | 45 | 1 | 6 |
| 2021Xu C miR-21 | 56 | 21 | 13 | 38 | N/R |
| 2021Xu C miR-210 | 59 | 16 | 10 | 43 | N/R |
| 2021Wang Q miR-378a-3p | 66 | 13 | 14 | 59 | N/R |
| 2021Wang D miR-1184 | 58 | 9 | 14 | 47 | 1.307 |
| 2021Trung N miR-146-3p | 85 | 25 | 45 | 57 | N/R |
| 2021Trung N miR-147b | 95 | 25 | 35 | 57 | N/R |
| 2021Trung N miR-155-5p | 81 | 22 | 49 | 60 | N/R |
| 2021Trung N miR-223-3p | 91 | 22 | 39 | 60 | N/R |
| 2021Sun B miR-486-5p | 95 | 16 | 13 | 85 | 1.3 |
| 2021Mao Y miR-455-5p | 72 | 13 | 18 | 75 | 1.2535 |
| 2021Liu J miR-381-3p | 80 | 17 | 22 | 88 | 0.699 |
| 2021Lin X miR-141-3p | 75 | 8 | 23 | 42 | 0.715 |
| 2021Li M miR-129-5p | 62 | 17 | 13 | 67 | 0.734 |
| 2020Zhao J miR-466 | 77 | 7 | 13 | 53 | N/R |
| 2020Yang Z miR-103 | 91 | 28 | 29 | 92 | 0.515 |
| 2020Yang Z miR-107 | 100 | 11 | 20 | 109 | 0.505 |
| 2020Wang J miR-25 | 70 | 2 | 12 | 28 | 0.39 |
| 2020Li H miR-150 | 17 | 2 | 13 | 28 | 2.27 |
| 2020Li H miR-107 | 29 | 8 | 1 | 22 | 1.43 |
| 2020Zhu X miR-125a | 88 | 62 | 32 | 58 | N/R |
| 2020Zhu X miR-125b | 59 | 24 | 61 | 96 | N/R |
| 2020Zhao D miR-125a | 93 | 39 | 57 | 111 | N/R |
| 2020Zhao D miR-125b | 135 | 63 | 15 | 87 | N/R |
| 2020Yang Y miR-125a | 94 | 30 | 8 | 70 | N/R |
| 2020Xu H miR-19b-3p | 88 | 14 | 15 | 84 | 0.817 |
| 2020Wang H miR-146a | 116 | 50 | 16 | 81 | N/R |
| 2020Wang H miR-451a | 86 | 12 | 12 | 53 | 1.465 |
| 2020Sun B miR-328 | 97 | 12 | 13 | 77 | 0.305 |
| 2020Salim R miR-101-3p | 42 | 5 | 8 | 25 | 0.936 |
| 2020Salim R miR-187 | 36 | 7 | 14 | 23 | 0.319 |
| 2020Salim R miR-21 | 32 | 10 | 18 | 20 | 0.732 |
| 2020Na L miR-21 | 208 | 83 | 11 | 136 | N/R |
| 2020Liu W miR-125a | 184 | 53 | 12 | 143 | N/R |
| 2020Liu G miR-181a | 85 | 8 | 17 | 42 | 0.625 |
| 2020Lin R miR-126-3p | 104 | 25 | 104 | 185 | 2.009 |
| 2020Dou H miR-155-5p | 142 | 11 | 61 | 89 | 2.098 |
| 2020Dou H miR-143 | 154 | 1 | 49 | 99 | 0.798 |
| 2020Chen W miR-146b | 90 | 15 | 14 | 85 | 0.865 |
| 2020Chen L miR-146a | 126 | 90 | 54 | 90 | N/R |
| 2020Chen L miR-146b | 148 | 31 | 32 | 149 | N/R |
| 2019Li W miR-21 | 61 | 31 | 22 | 19 | N/R |
| 2019Zhang W miR-7110-5p | 37 | 5 | 7 | 47 | 4.41 |
| 2019Zhang W miR-223-3p | 37 | 0 | 7 | 52 | 2.759 |
| 2019Karam R miR-146a | 48 | 26 | 7 | 34 | 0.5 |
| 2019Guo H miR-495 | 95 | 17 | 10 | 83 | 0.655 |
| 2018Li J miR-142-3p | 28 | 6 | 13 | 14 | N/R |
| 2018Wu X miR-223-3p | 107 | 24 | 80 | 162 | N/R |
| 2018Rahmel T miR-122 | 63 | 1 | 45 | 19 | 0.004 |
| 2018Chen C miR-126-3p | 34 | 2 | 26 | 23 | N/R |
| 2018Chao L miR-155-5p | 89 | 7 | 16 | 28 | 1.64 |
| 2018Chao L miR-133a-3p | 103 | 16 | 2 | 19 | 0.82 |
| 2017Liu Z miR-122a | 77 | 7 | 26 | 23 | 1.96 |
| 2017Liu Z miR-146a | 72 | 9 | 31 | 21 | 2 |
| 2017Liu Z miR-155a | 72 | 8 | 31 | 22 | 1.89 |
| 2017Lin H miR-15b | 52 | 5 | 30 | 17 | 0.0602 |
| 2017Lin H miR-210 | 38 | 1 | 44 | 21 | 0.0175 |
| 2017Lin H miR-486-5p | 69 | 3 | 13 | 19 | 2.7606 |
| 2016Han Y miR-143 | 81 | 8 | 22 | 87 | 15.9 |
| 2015Yao L miR-25 | 51 | 7 | 19 | 23 | N/R |
| 2015Wang X miR-15a | 29 | 1 | 17 | 40 | N/R |
| 2015Wang X miR-16-5p | 34 | 2 | 12 | 39 | N/R |
| 2015Wang X miR-15b | 23 | 10 | 23 | 31 | N/R |
| 2015Wang X miR-223-3p | 31 | 16 | 15 | 25 | N/R |
| 2013Deng J miR-122 | 50 | 11 | 2 | 12 | 0.71 |

TP = true positive, FP = false positive, FN = false negative, TN = true negative, miR = mircoRNA, N/R = not report.
