# Supplementary material for: Diagnostic significance of microRNAs in sepsis
Source: PLoS One. 2023 Feb 22;18(2):e0279726. doi: 10.1371/journal.pone.0279726 (PMC9946237; doi:10.1371/journal.pone.0279726)
Supplement: S1 Appendix — (DOCX) [file pone.0279726.s003.docx]

2022-5-12

Search strategies for EMBASE (A), the Cochrane Central Register of Controlled Trials (B), and China National Knowledge Infrastructure (C).

A: Embase database was searched as follows: #1 'sepsis' OR 'pyemia' OR 'septicemia', #2 'MicroRNAs' OR 'MicroRNA' OR 'miRNAs' OR 'miRNA', #1 AND #2.

| Embase | | |
| --- | --- | --- |
| 1 | ('sepsis' or 'pyemia' or 'septicemia').mp. [mp=title, abstract, heading word, drug trade name, original title, device manufacturer, drug manufacturer, device trade name, keyword, floating subheading word, candidate term word] | 362428 |
| 2 | ('MicroRNAs' or 'MicroRNA' or 'miRNAs' or 'miRNA').mp. [mp=title, abstract, heading word, drug trade name, original title, device manufacturer, drug manufacturer, device trade name, keyword, floating subheading word, candidate term word] | 218501 |
| 3 | #1 AND #2 | 1612 |

## B: the Cochrane Central Register of Controlled Trials database was searched as follows:  (‘sepsis’ OR ‘pyemia’ OR ‘septicemia’) AND (‘MicroRNAs’ OR ‘MicroRNA’ OR ‘miRNAs’ OR ‘miRNA’) in Title Abstract Keyword

| the Cochrane Central Register of Controlled Trials | | |
| --- | --- | --- |
| 1 | (‘sepsis’ OR ‘pyemia’ OR ‘septicemia’) in Title Abstract Keyword | 12622 |
| 2 | (‘MicroRNAs’ OR ‘MicroRNA’ OR ‘miRNAs’ OR ‘miRNA’) in Title Abstract Keyword | 1233 |
| 3 | #1 AND #2 | 10 |

## C: China National Knowledge Infrastructure database was searched as follows:  (sepsis OR pyemia OR septicemia) AND (MicroRNAs OR MicroRNA OR miRNAs OR miRNA) in Title Abstract Keyword.

| China National Knowledge Infrastructure database | | |
| --- | --- | --- |
| 1 | (sepsis OR pyemia OR septicemia) in Title Abstract Keyword | 235474 |
| 2 | (MicroRNAs OR MicroRNA OR miRNAs OR miRNA) in Title Abstract Keyword | 203017 |
| 3 | #1 AND #2 | 926 |
