# Supplementary material for: Diagnostic significance of microRNAs in sepsis
Source: PLoS One. 2023 Feb 22;18(2):e0279726. doi: 10.1371/journal.pone.0279726 (PMC9946237; doi:10.1371/journal.pone.0279726)
Supplement: S1 Fig — (A) Methodological quality graph; (B) Methodological quality summary. (PDF) [file pone.0279726.s004.pdf]

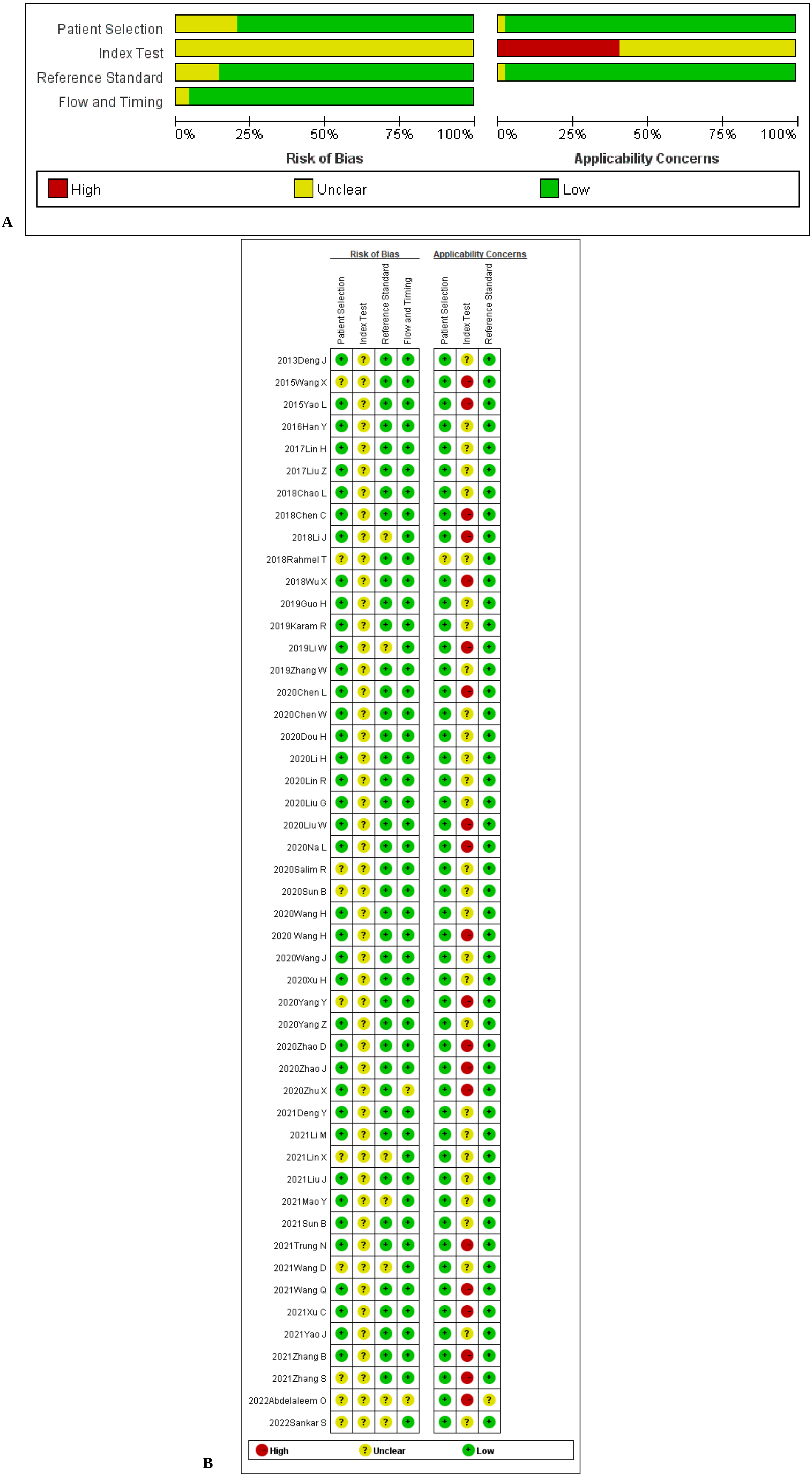
A
Patient Selection
Index Test
Reference Standard
Flow and Timing
0%
25%
50%
75%
100%
Risk of Bias
Applicability Concerns
High
Unclear
Low
B
Risk of Bias
Applicability Concerns
Patient Selection
Index Test
Reference Standard
Flow and Timing
2013Deng J
2015Wang X
2015Yao L
2016Han Y
2017Lin H
2017Liu Z
2018Chao L
2018Chen C
2018Li J
2018Rahmel T
2018Wu X
2019Guo H
2019Karam R
2019Li W
2019Zhang W
2020Chen L
2020Chen W
2020Dou H
2020Li H
2020Lin R
2020Liu G
2020Liu W
2020Na L
2020Salim R
2020Sun B
2020Wang H
2020 Wang H
2020Wang J
2020Xu H
2020Yang Y
2020Yang Z
2020Zhao D
2020Zhao J
2020Zhu X
2021Deng Y
2021Li M
2021Lin X
2021Liu J
2021Mao Y
2021Sun B
2021Trung N
2021Wang D
2021Wang Q
2021Xu C
2021Yao J
2021Zhang B
2021Zhang S
2022Abdelaleem O
2022Sankar S
High
Unclear
Low
