# Supplementary figures and images for: Diagnostic significance of microRNAs in sepsis
Source: PLoS One. 2023 Feb 22;18(2):e0279726. doi: 10.1371/journal.pone.0279726 (PMC9946237; doi:10.1371/journal.pone.0279726)

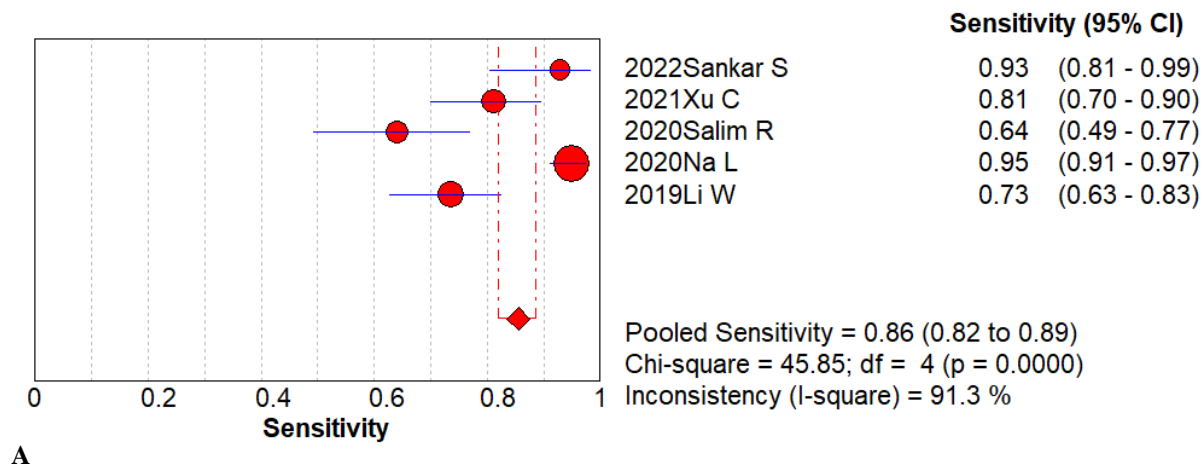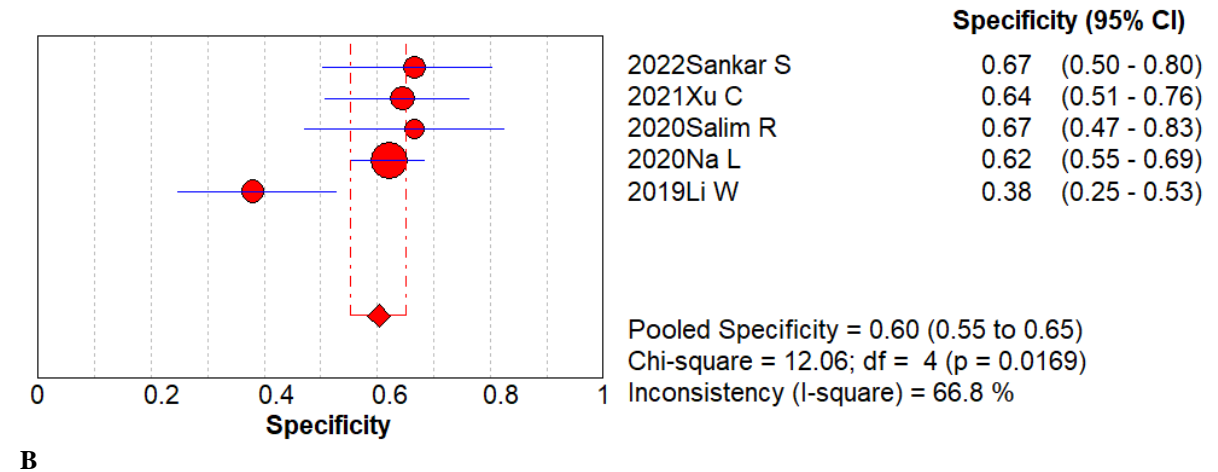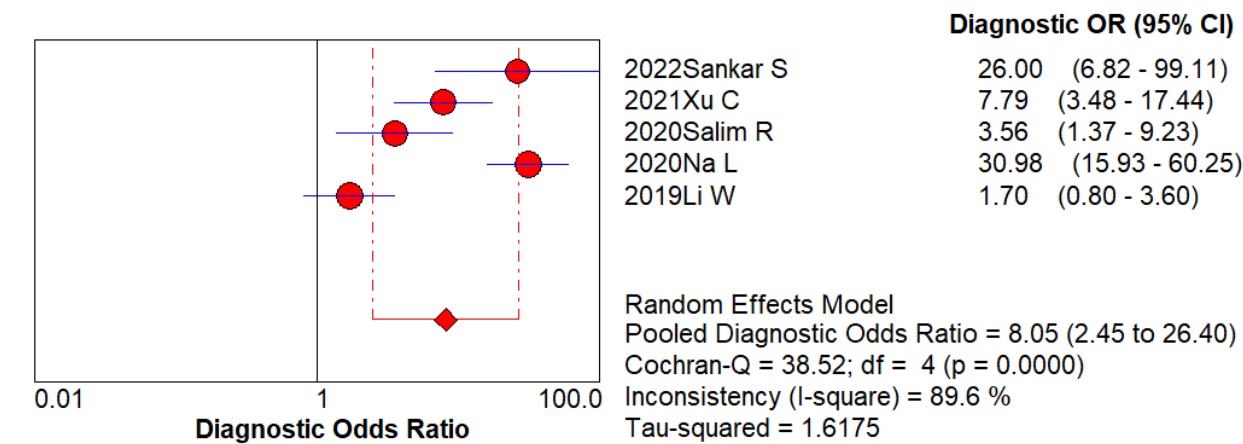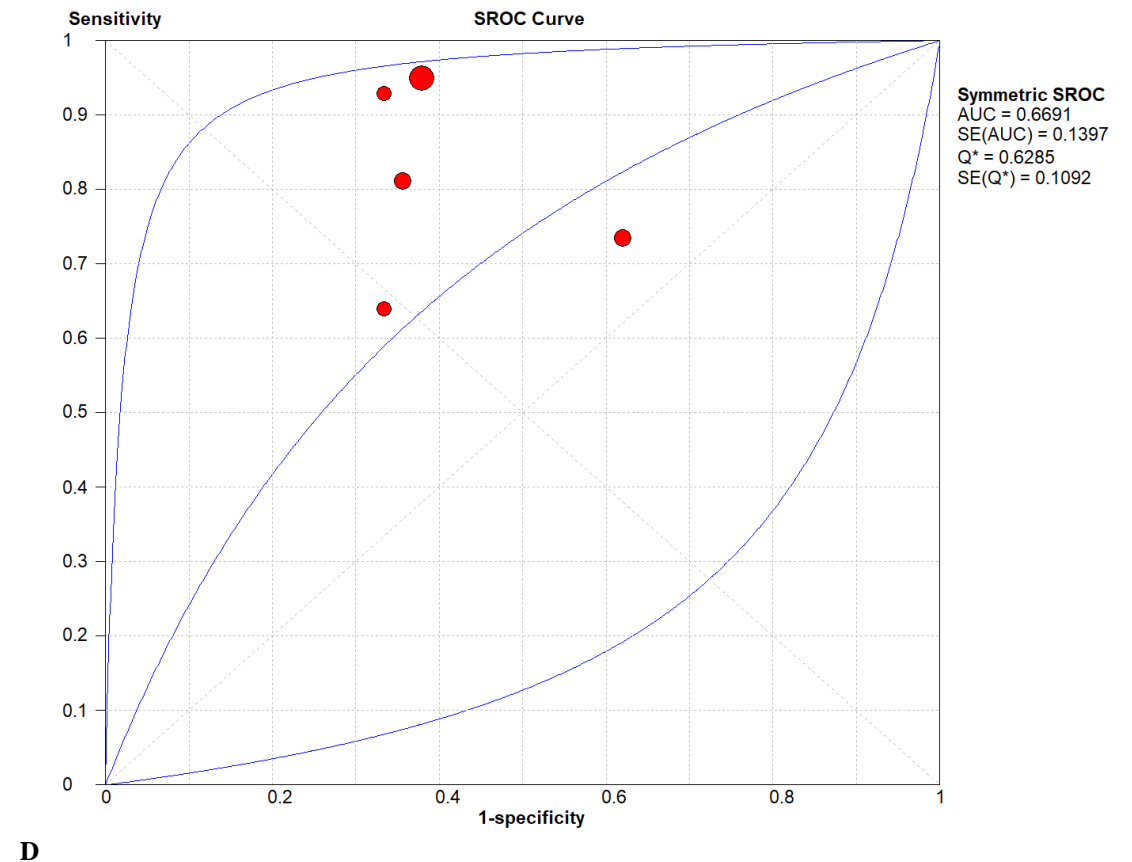

Supplement: S2 Fig — (A) Pooled sensitivity. (B) Pooled specificity. (C) Overall DOR. (D) The SROCs for all datasets. (PDF) [file pone.0279726.s005.pdf]

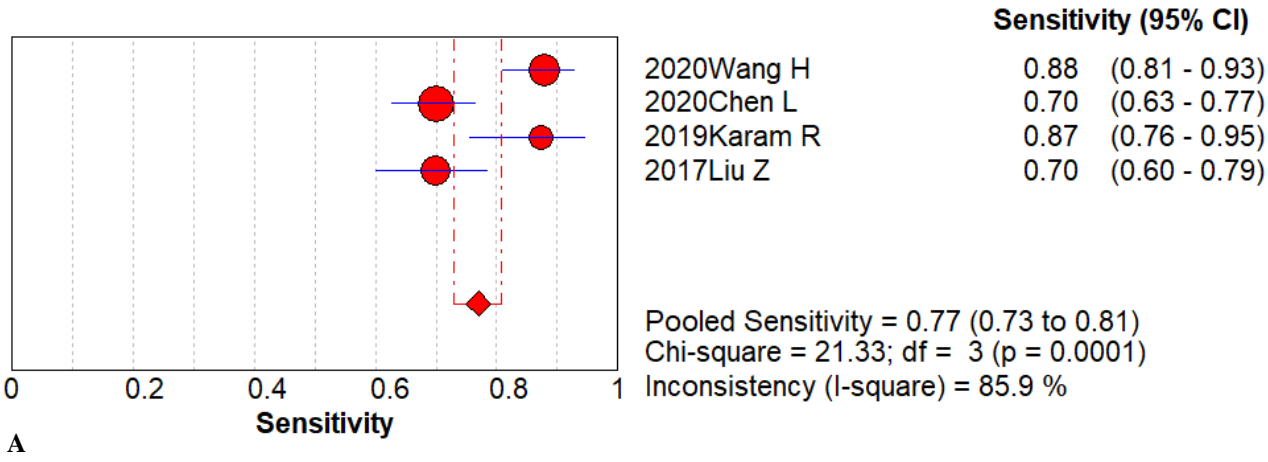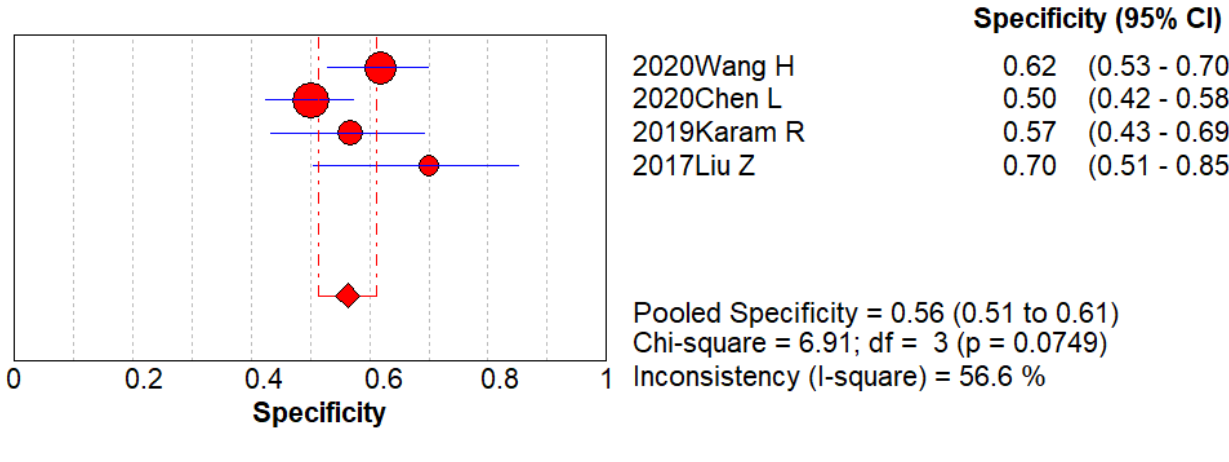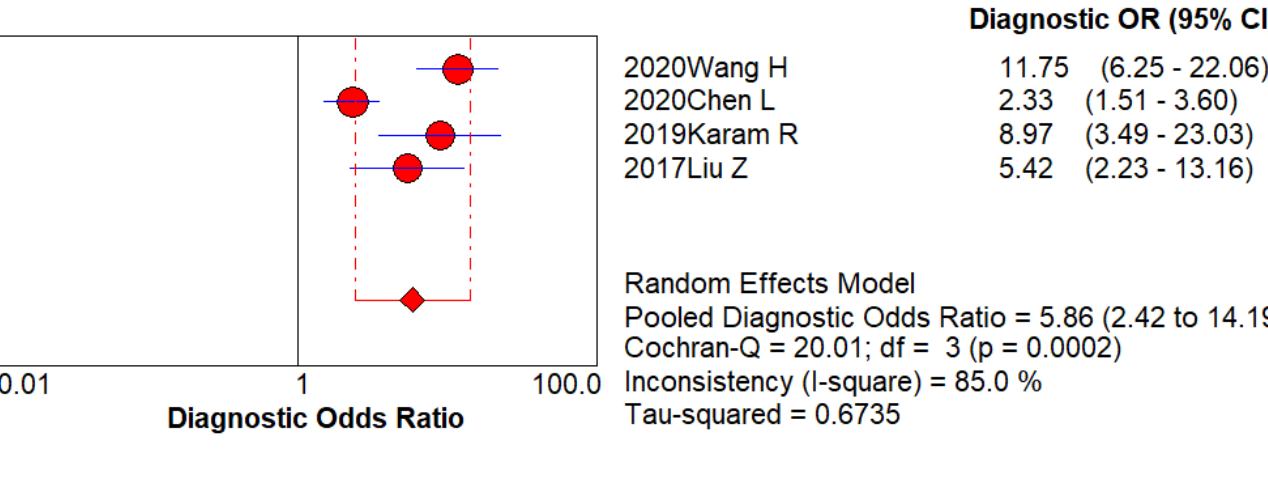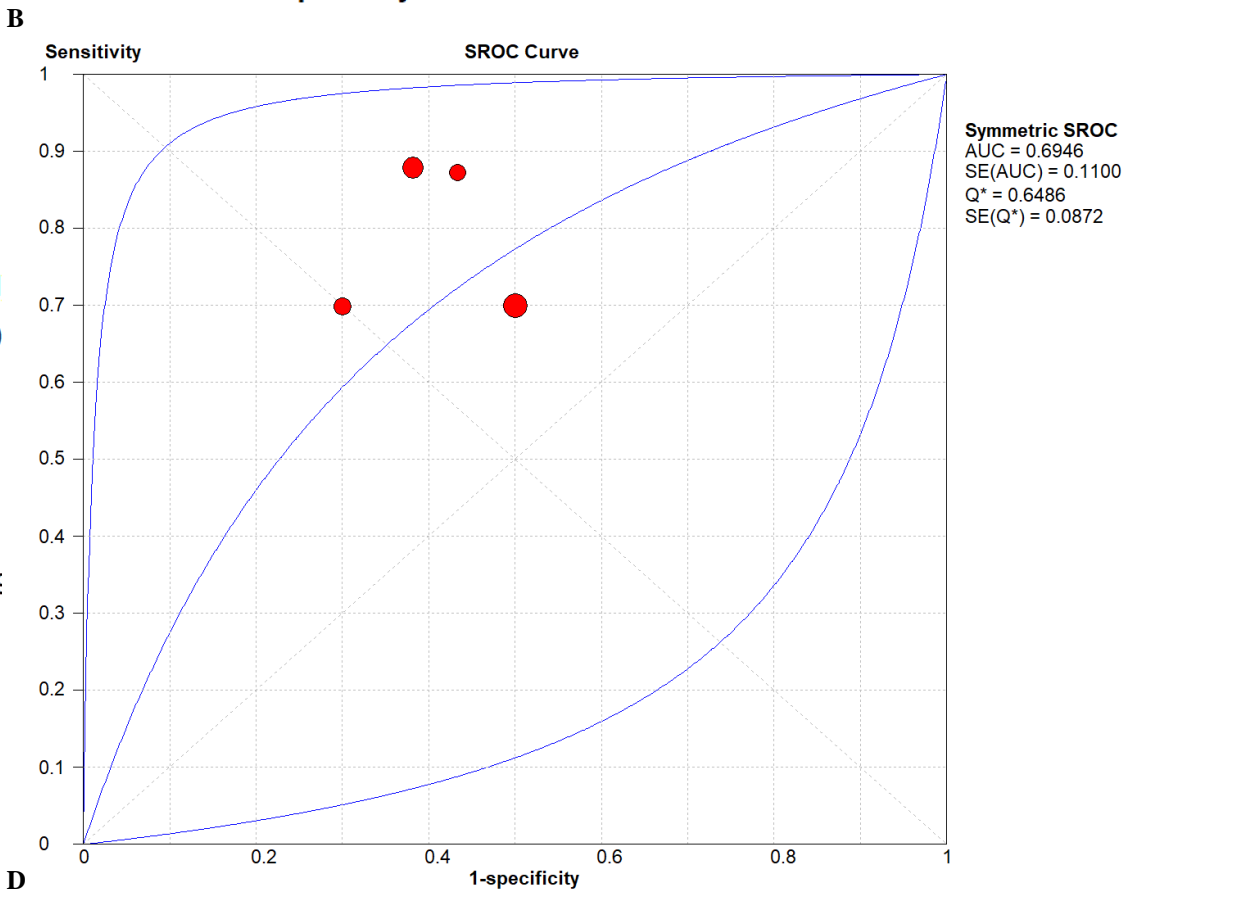

Supplement: S4 Fig — (A) Pooled sensitivity. (B) Pooled specificity. (C) Overall DOR. (D) The SROCs for all datasets. (PDF) [file pone.0279726.s007.pdf]

A

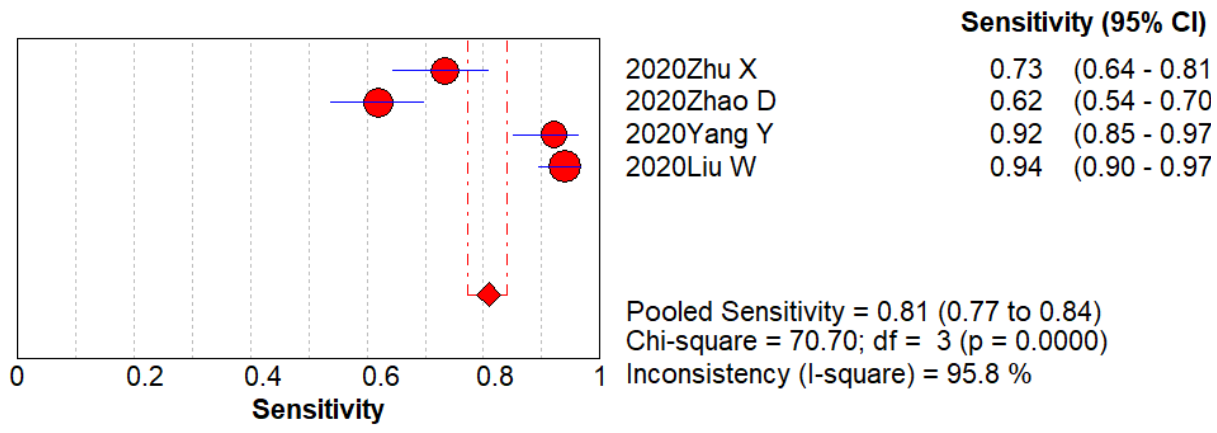

B

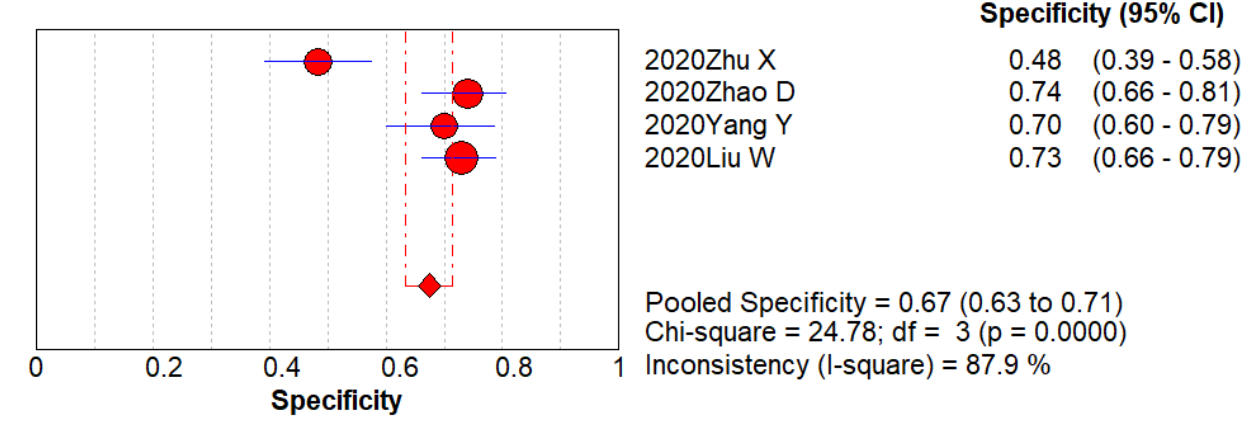

C

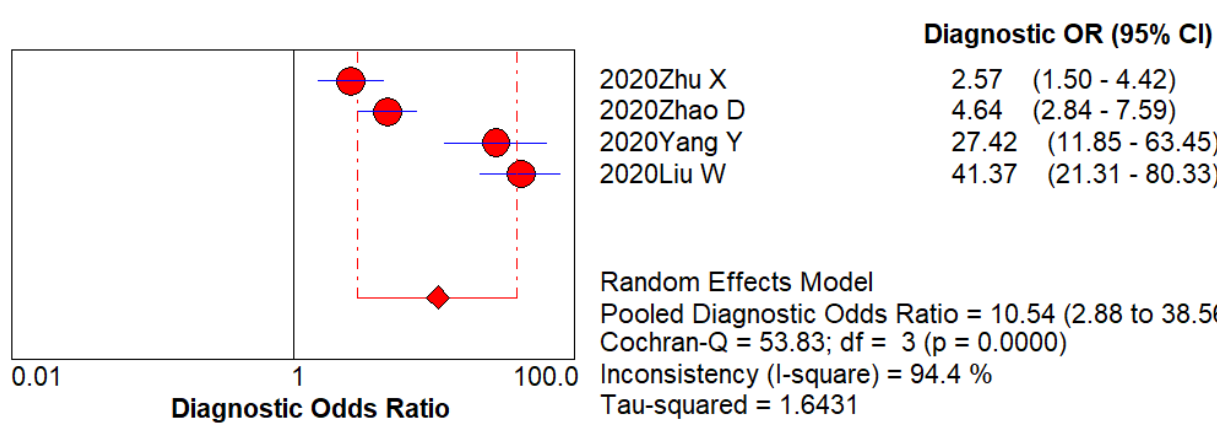

D

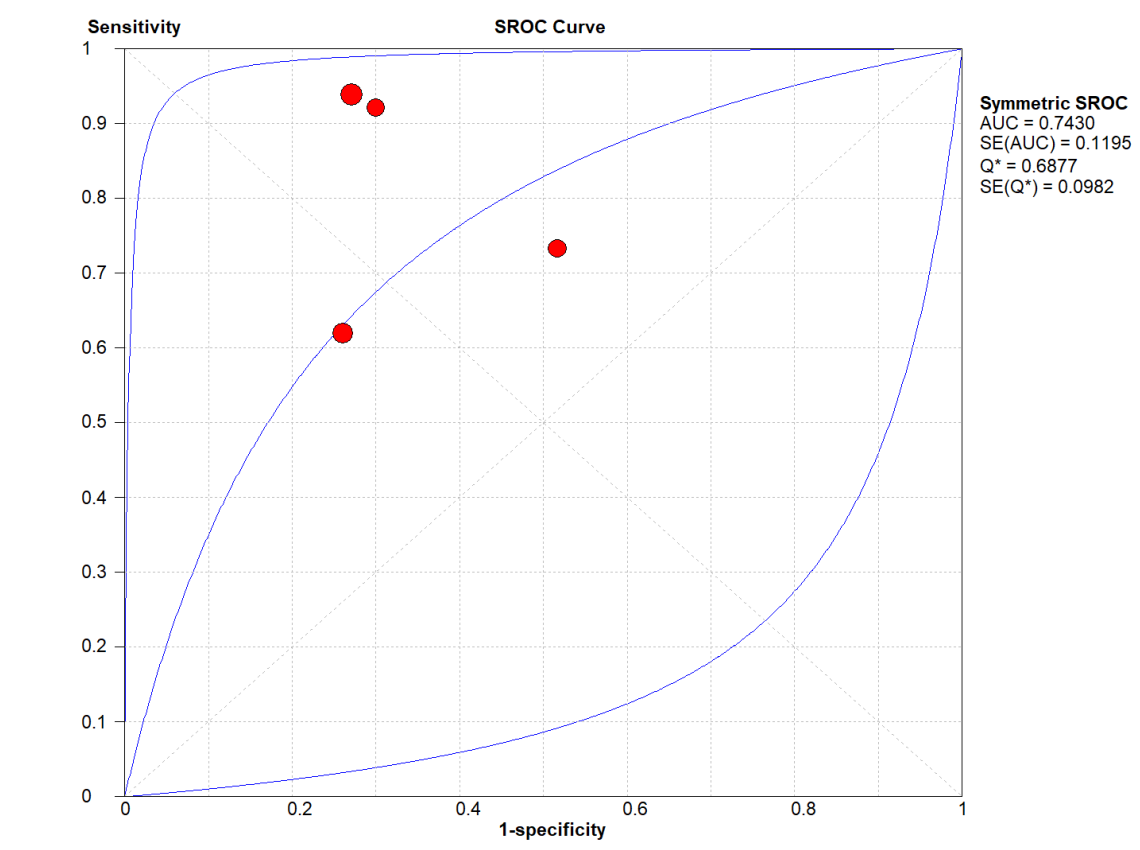

Supplement: S5 Fig — (A) Pooled sensitivity. (B) Pooled specificity. (C) Overall DOR. (D) The SROCs for all datasets. (PDF) [file pone.0279726.s008.pdf]

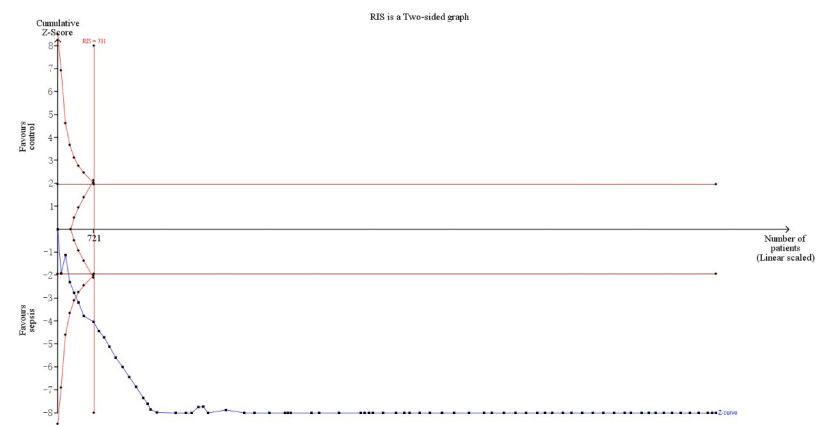

**A**

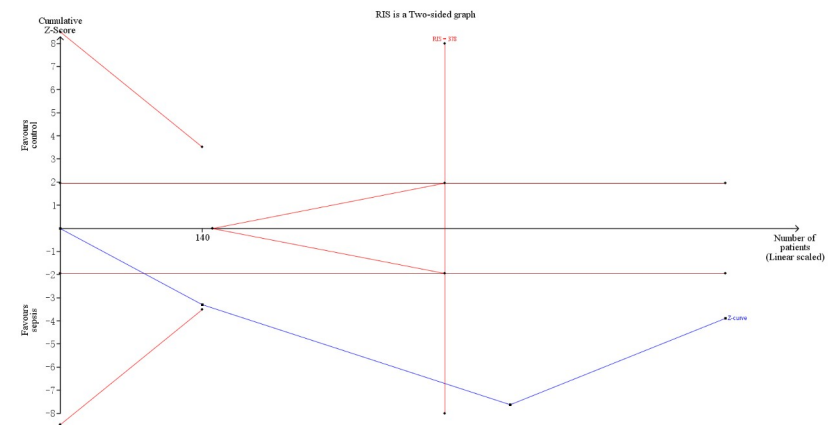

**B**

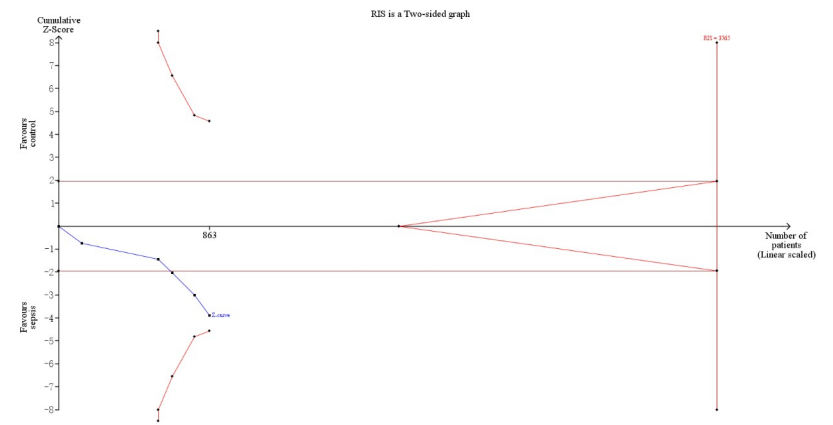

**C**

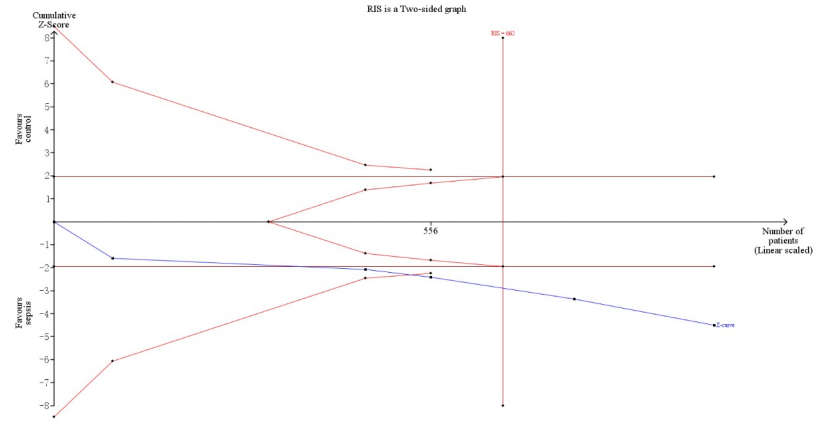

**D**

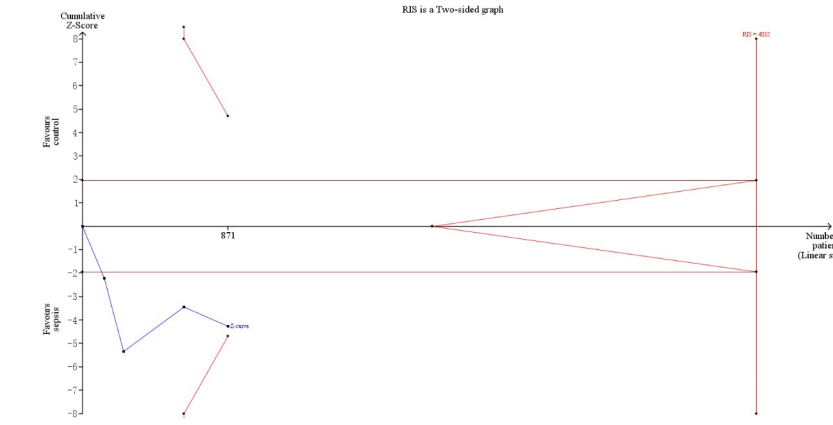

**E**

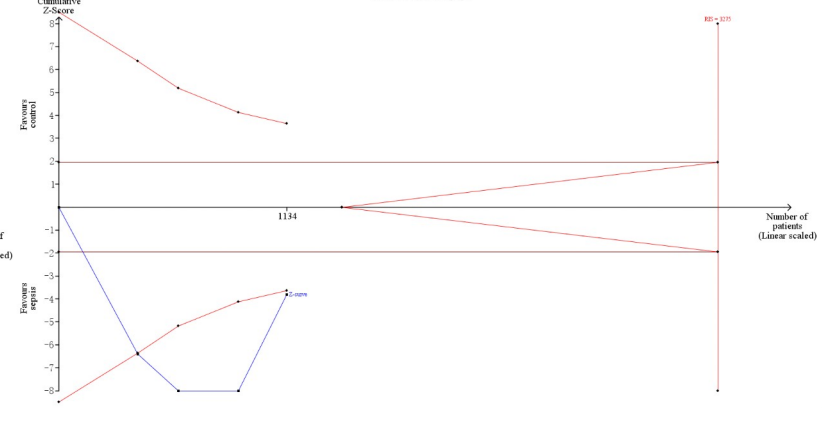

**F**

Supplement: S6 Fig — (A) TSA plot of TmiR for sepsis diagnosis. (B) TSA plot of miR-155-5p for sepsis diagnosis. (C) TSA plot of miR-21 for sepsis diagnosis. (D) TSA plot of miR-223-3p for sepsis diagnosis. (E) TSA plot of miR-146a for sepsis diagnosis. (F) TSA plot of miR-125a for sepsis diagnosis. (PDF) [file pone.0279726.s009.pdf]
