# Supplementary material for: Diagnostic significance of microRNAs in sepsis
Source: PLoS One. 2023 Feb 22;18(2):e0279726. doi: 10.1371/journal.pone.0279726 (PMC9946237; doi:10.1371/journal.pone.0279726)
Supplement: S3 Fig — (A) Pooled sensitivity. (B) Pooled specificity. (C) Overall DOR. (D) The SROCs for all datasets. (PDF) [file pone.0279726.s006.pdf]

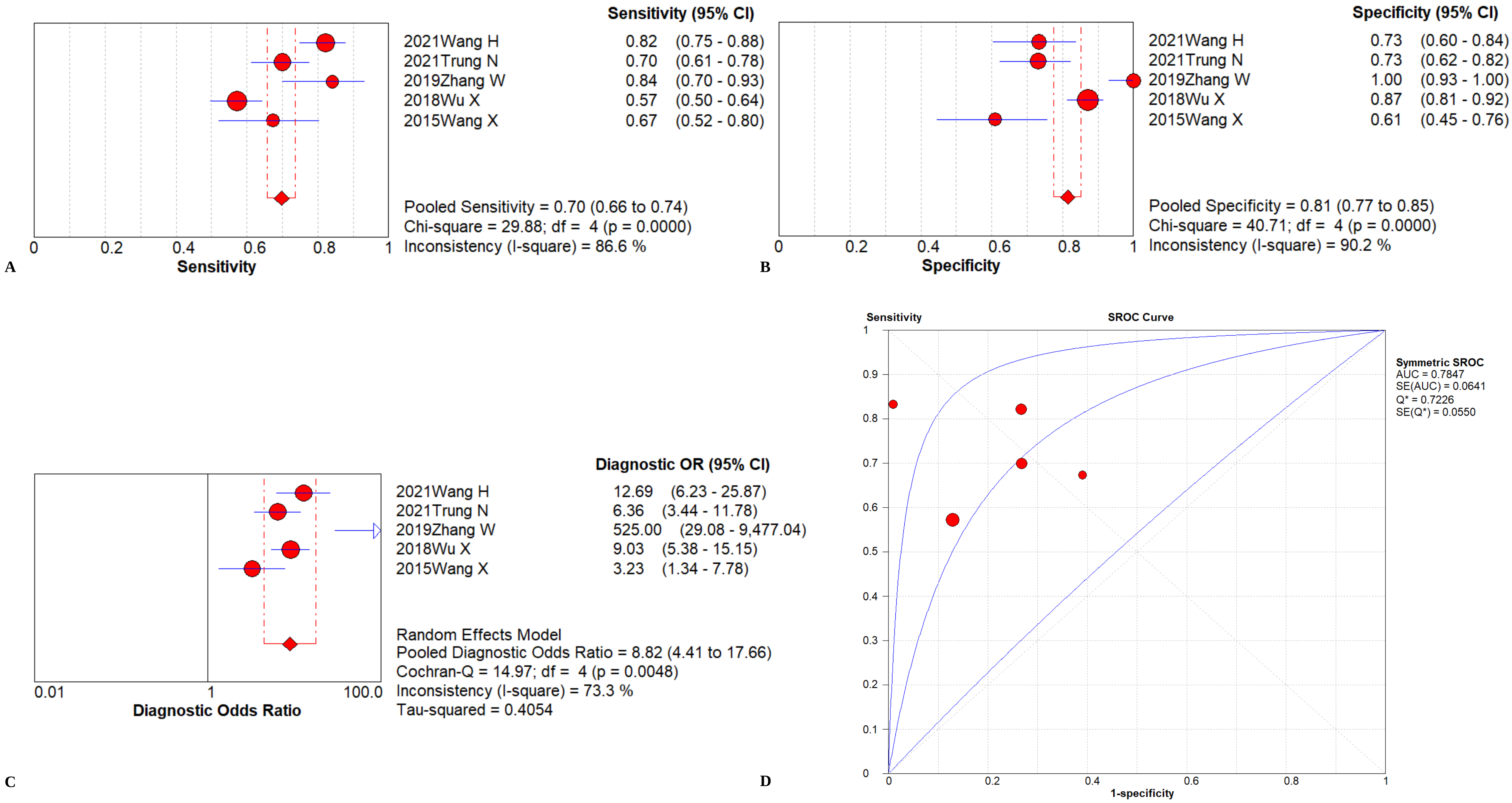

Sensitivity (95% CI)
2021Wang H 0.82 (0.75 - 0.88)
2021Trung N 0.70 (0.61 - 0.78)
2019Zhang W 0.84 (0.70 - 0.93)
2018Wu X 0.57 (0.50 - 0.64)
2015Wang X 0.67 (0.52 - 0.80)
Pooled Sensitivity = 0.70 (0.66 to 0.74)
Chi-square = 29.88; df = 4 (p = 0.0000)
Inconsistency (I-square) = 86.6 %
0 0.2 0.4 0.6 0.8 1
Sensitivity
A
Specificity (95% CI)
2021Wang H 0.73 (0.60 - 0.84)
2021Trung N 0.73 (0.62 - 0.82)
2019Zhang W 1.00 (0.93 - 1.00)
2018Wu X 0.87 (0.81 - 0.92)
2015Wang X 0.61 (0.45 - 0.76)
Pooled Specificity = 0.81 (0.77 to 0.85)
Chi-square = 40.71; df = 4 (p = 0.0000)
Inconsistency (I-square) = 90.2 %
0 0.2 0.4 0.6 0.8 1
Specificity
B
Diagnostic OR (95% CI)
2021Wang H 12.69 (6.23 - 25.87)
2021Trung N 6.36 (3.44 - 11.78)
2019Zhang W 525.00 (29.08 - 9,477.04)
2018Wu X 9.03 (5.38 - 15.15)
2015Wang X 3.23 (1.34 - 7.78)
Random Effects Model
Pooled Diagnostic Odds Ratio = 8.82 (4.41 to 17.66)
Cochran-Q = 14.97; df = 4 (p = 0.0048)
Inconsistency (I-square) = 73.3 %
Tau-squared = 0.4054
0.01 1 100.0
Diagnostic Odds Ratio
C
Sensitivity
SROC Curve
1 0.9 0.8 0.7 0.6 0.5 0.4 0.3 0.2 0.1 0
Symmetric SROC
AUC = 0.7847
SE(AUC) = 0.0641
Q* = 0.7226
SE(Q*) = 0.0550
0 0.2 0.4 0.6 0.8 1
1-specificity
D
